# Structure of the Cellulose Synthase Complex of *Gluconacetobacter hansenii* at 23.4 Å Resolution

**DOI:** 10.1371/journal.pone.0155886

**Published:** 2016-05-23

**Authors:** Juan Du, Venkata Vepachedu, Sung Hyun Cho, Manish Kumar, B. Tracy Nixon

**Affiliations:** 1 Department of Biochemistry & Molecular Biology, The Pennsylvania State University, University Park, PA, 16802, United States of America; 2 Department of Chemical Engineering, The Pennsylvania State University, University Park, PA, 16802, United States of America; Chang-Gung University, TAIWAN

## Abstract

Bacterial crystalline cellulose is used in biomedical and industrial applications, but the molecular mechanisms of synthesis are unclear. Unlike most bacteria, which make non-crystalline cellulose, *Gluconacetobacter hansenii* extrudes profuse amounts of crystalline cellulose. Its cellulose synthase (AcsA) exists as a complex with accessory protein AcsB, forming a 'terminal complex' (TC) that has been visualized by freeze-fracture TEM at the base of ribbons of crystalline cellulose. The catalytic AcsAB complex is embedded in the cytoplasmic membrane. The C-terminal portion of AcsC is predicted to form a translocation channel in the outer membrane, with the rest of AcsC possibly interacting with AcsD in the periplasm. It is thus believed that synthesis from an organized array of TCs coordinated with extrusion by AcsC and AcsD enable this bacterium to make crystalline cellulose. The only structural data that exist for this system are the above mentioned freeze-fracture TEM images, fluorescence microscopy images revealing that TCs align in a row, a crystal structure of AcsD bound to cellopentaose, and a crystal structure of PilZ domain of AcsA. Here we advance our understanding of the structural basis for crystalline cellulose production by bacterial cellulose synthase by determining a negative stain structure resolved to 23.4 Å for highly purified AcsAB complex that catalyzed incorporation of UDP-glucose into β-1,4-glucan chains, and responded to the presence of allosteric activator cyclic diguanylate. Although the AcsAB complex was functional in vitro, the synthesized cellulose was not visible in TEM. The negative stain structure revealed that AcsAB is very similar to that of the BcsAB synthase of *Rhodobacter sphaeroides*, a non-crystalline cellulose producing bacterium. The results indicate that the crystalline cellulose producing and non-crystalline cellulose producing bacteria share conserved catalytic and membrane translocation components, and support the hypothesis that it is the extrusion mechanism and order in linearly arrayed TCs that enables production of crystalline cellulose.

## Introduction

Cellulose is the most abundant polysaccharide on earth. Cellulose microfibrils are composed of long polymeric chains of β-1,4 linked D-glucose. Over the past decades, cellulose has attracted increasing interest. Cellulose is the major structural component of plant cell walls and also important in many bacterial biofilms [1]. The cellulose in plant biomass is valued as a resource for sustainable biofuels [2, 3], and bacterial cellulose has been used to meet important industrial and biomedical needs due to its high purity, high degree of polymerization, high crystallinity, high water content and high mechanical stability [4–7].

Among cellulose-producing bacteria, some of which include Gluconacetobacter (formerly Acetobacter [8] and recently renamed to Komagataeibacter [9, 10]), Agrobacterium, Aerobacter, Achromobacter, Azotobacter, Rhizobium, Sarcina, Salmonella, Escherichia, Pseudomonas and Alcaligenes [11–13], the gram-negative *G*. *hansenii* has been subjected to intensive study as a model organism for cellulose biosynthesis. Unique among bacteria, Gluconacetobacter synthesize and extrude profuse amounts of crystalline cellulose of type I structure to help float microbial mats to the air water interface, perhaps to facilitate contact with oxygen [14–16]. The cellulose synthase machinery in *G*. *hansenii* was observed as a Terminal Complex (TC) arranged in linear rows by freeze-fracture transmission electron microscopy and immunogold labeling [17, 18]. Fluorescent protein tagged TC components (AcsD and CcpAx) were localized in a line longitudinally along with the cell [19]. The first cellulose synthase gene to be identified was *acsA* of *G*. *hansenii*, and a number of studies have shown that the synthase works optimally when supplemented with the products of the genes *acsB*, *acsC* and *acsD* [20–22]. AcsA, the catalytic subunit, is an 83 kDa polypeptide localized in the cytoplasmic membrane that is able to transfer glucose to the growing glucan chain [23–26]. AcsA contains a catalytic domain in an intracellular loop and a regulatory PilZ domain in its carboxy terminus. The PilZ domain binds the cellulose synthesis allosteric activator cyclic diguanylate [27–31]. AcsB was identified via product entrapment as a 93 kDa polypeptide that co-purified with AcsA [25, 32, 33]. AcsB is a periplasmic protein and by homology to BcsB of *R*. *sphaeroides* it is believed to be anchored to the cytoplasmic membrane with a single C-terminal transmembrane helix (TMH) [32]. Although all functions of BcsB, and by analogy AcsB, have not been demonstrated, the C-terminal helix has been shown to have three functions. It anchors BcsB to the membrane, binds BcsB to the synthase protein, and is essential for the latter's synthesis of cellulose [34]. The homology of AcsC with bacterial macromolecule secretion systems suggests it may form a transmembrane pore [21]. AcsC is thus believed to facilitate the transport of glucan chains outside the cell where they form microfibrils. Note that many bacteria that synthesize non-crystalline cellulose don't have recognizable *acsC* genes [35]. *G*. *hansenii* cells lacking AcsD still produce cellulose but with a reduced yield, modified morphology and lowered crystallinity, indicating a role for AcsD in crystallization of cellulose [21, 36]. AcsD is a soluble protein present in the periplasmic space [37], and its crystal structure revealed a homomeric octamer that forms a 2x4-ring structure with an apparent capacity to hold four β-glucan chains in close proximity [36].

Of all functional cellulose synthase proteins, only that of *R*. *sphaeroides* BcsAB complex has been structurally defined, in that case by X-ray crystallography. This ground breaking work advanced our understanding of the structural basis of cellulose synthesis at atomic resolution [38, 39]. However, neither the highly purified complex nor Rhodobacter itself makes crystalline cellulose [34]. It remains unknown if there are structural features of the *G*. *hansenii* synthase that contribute to crystal formation of the produced cellulose, or if crystallinity results solely from appropriate presentation of several synthases in the linear array of TCs to the AcsD/AcsC export machinery. To address this question, it is important to obtain structure of the cellulose synthase complex of *G*. *hansenii*. Here we report the systematic optimization of purification of cellulose synthase AcsAB from *G*. *hansenii*. The yields were insufficient for crystallization trials, but adequate to demonstrate production of apparently non-crystalline cellulose and negative stain structure determination by electron microscopy (this structural method works well for smaller protein complexes). The negative stain images were used to construct a 3D volume defined at 23.4 Å resolution. We discuss how this structure relates to that of BcsAB, underscoring an apparent need for linear arrangement of AcsAB for *G*. *hansenii* to make crystalline cellulose.

## Results

### Expression of the TC Components AcsAB and AcsC

**Fig 1:**
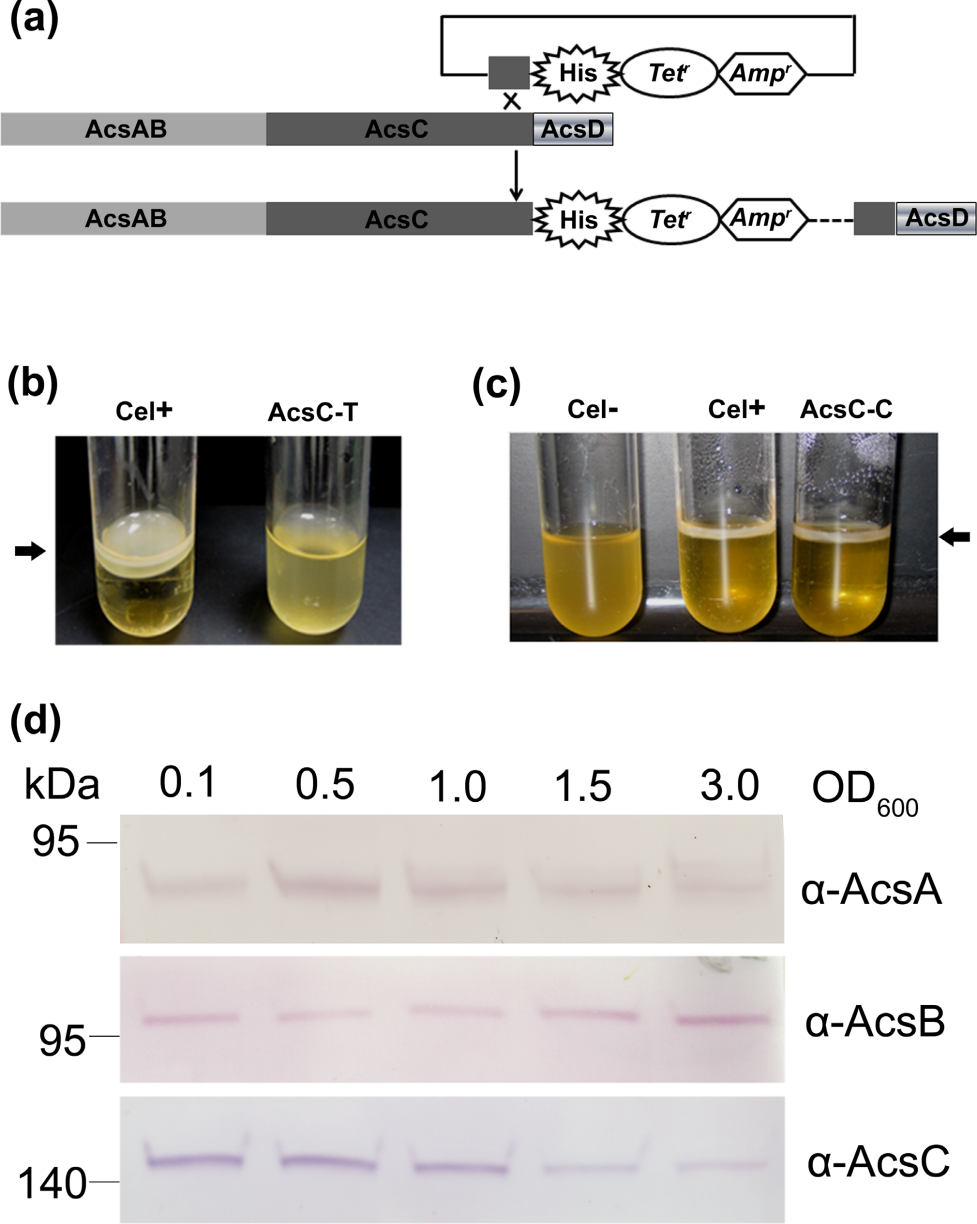
Expression affinity tagged cellulose synthase in *G*. *hansenii* via homologous recombination. (a) Strategy for constructing C-terminal His tagged AcsC by homologous recombination. AcsAB, AcsC and AcsD represent the coding sequences for the corresponding genes. "His" is a dodeca-histidine tag. Tet^r^ and Amp^r^ indicate the coding sequence for tetracycline and ampicilin resistance gene, respectively. (b) The Cel^+^ strain engineered to express AcsC-T was unable to produce cellulose in static culture, although its parent produced cellulose at the air-media interface [41]. (c) Static cultures of the Cel^+^ strain engineered to express AcsC-C made cellulose pellicle like its parent Cel^+^, while a stable non-cellulose-producing strain (Cel^-^) did not [41]. (d) Expression profiling of AcsA, AcsB and AcsC in AcsC-C strain. The cells were harvested in the indicated OD values. Polyclonal antibodies raised and purified against synthetic peptides of AcsA (α-AcsA), AcsB (α-AcsB) and AcsC (α-AcsC) [37] were used, respectively.

Our first strategy to express affinity tagged TC was to integrate a histidine tag in the C terminus of AcsC by homologous recombination (Fig 1A). Inserting the tag at the C-terminal end of published sequence for *acsC* in *G*. *hansenii* ATCC 23769 (gene GXY_04282 [40, 41]) caused the engineered cells to fail to make cellulose (Fig 1B). Secondary structure predictions of AcsC suggest a fairly large domain made of seventeen tetratricopeptide repeats (TPRs), followed by a β-barrel at the C-terminus (S1 Fig). However, the β-barrel predicted from published sequence appeared truncated (so we denote that as AcsC-T), suggesting a mistake had been made assigning its coding region in the deposited sequence (S1 Fig). Re-sequencing that region revealed a frame shift that caused a premature termination in the published sequence. After sequence correction, the peptide was elongated 107 amino acid residues and these are predicted to complete the β-barrel structure (S1 Fig, AcsC-C). When the tag was moved to the C-terminal end of AcsC-C, the strain was able to make crystalline cellulose (Fig 1C) and produce AcsC (also, AcsA and AcsB) protein as determined by Western blotting using polyclonal antibody against fragments of either AcsA, AcsB or AcsC (Fig 1D). Cells harvested at OD600_nm_ equal to 1.0 yielded pure AcsC-His12 but there was no co-purification of AcsAB (data not shown). The amount of AcsC-His produced was extremely low, unsuitable for structural studies and failed to provide a handle for purifying a complete TC.

**Fig 2:**
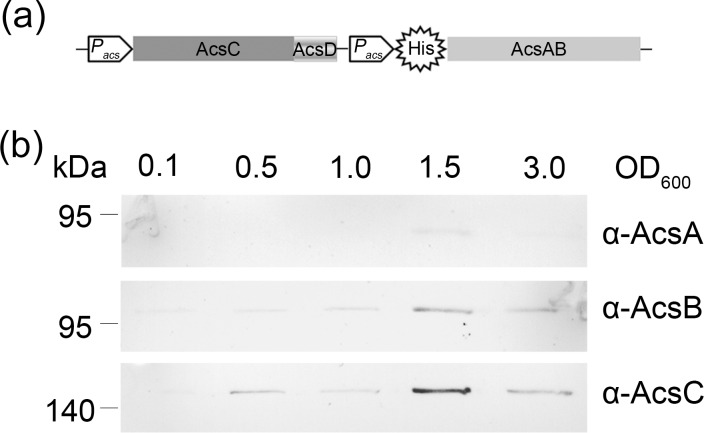
Expression of affinity tagged cellulose synthase carried by plasmid in *G*. *hansenii*. (a) Schematic of plasmid expressing AcsAB, AcsC and AcsD. *P*_*acs*_ promoter is the original promoter of cellulose synthase operon in the strain ATCC23769. "His" is a dodeca-histidine tag fused in the N-terminal of AcsAB. AcsAB, AcsC and AcsD indicate the coding sequences of AcsAB, AcsC and AcsD, respectively. (b) Western blot of cells transformed with pUCD2CDHisAcsAB and harvested at the indicated OD values.

Failing to express sufficient affinity tagged TC by homologous recombination of C-terminally tagged AcsC, we developed a plasmid-based, homologous expression system to produce N-terminally tagged AcsAB. Unlike bacterial synthase proteins BcsA and BcsB, which are encoded on distinct genes, AcsA and AcsB of *G*. *hansenii* are fused into one larger polypeptide that is proteolytically processed to generate the separate proteins of the AcsAB complex [33]. The endogenous promoter of the *acs* operon was used to drive the expression of the normally fused *acsAB* gene but with insertion of an N-terminal dodeca-histidine tag. The same promoter was also placed in the front of the *acsCD* genes to activate their expression (Fig 2A). The whole transcriptional cassette encoding AcsAB, AcsC and AcsD was inserted into the plasmid pUCD2 and stably transformed into wild type *G*. *hansenii*. Western blotting of transformed cells revealed the expression of AcsA, AcsB and AcsC gradually increased until OD600_nm_ reached 1.5 and then decreased over the time (Fig 2B). The cells were thus cultured to an OD600_nm_ of 1.5 and harvested for protein purification. Note that when expressing these proteins, copious amounts (0.2 g/liter) of cellulase (Worthington) had to be added to the culture medium to remove cellulose to facilitate large scale fermentations.

### Detergent Screening for the Purification of AcsAB Complex

**Fig 3:**
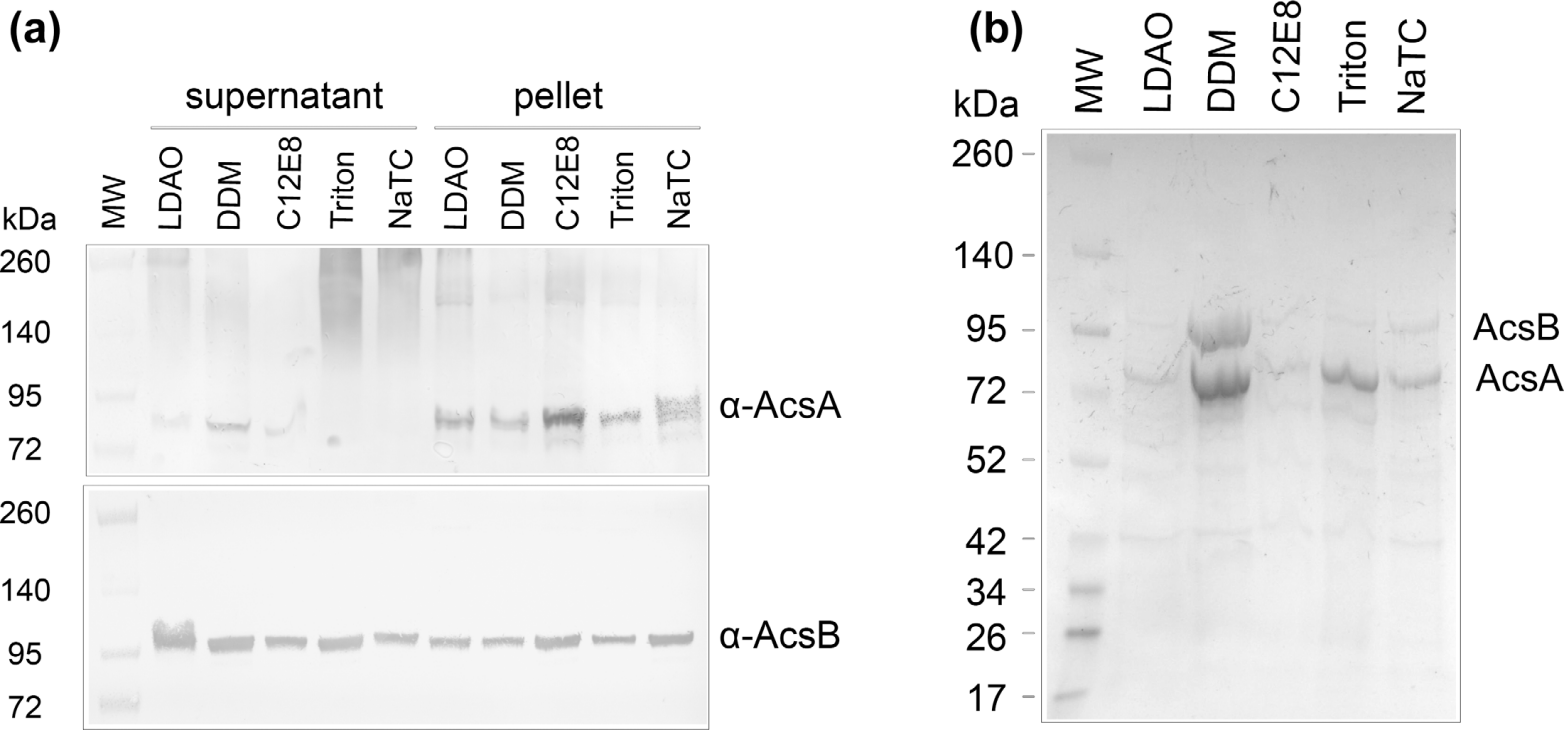
Detergent screening for solubilization of AcsAB from membrane. The membranes of cells transformed with pUCD2CDHisAcsAB were incubated with the indicated detergent for 60 min at 4°C followed by ultra-centrifugation. (a) Western blots of supernatant and pellet fractions using antibody against AcsA and AcsB, respectively. (b) SDS-PAGE and coomassie staining of Co-NTA purified protein from the supernatant solubilized by the indicated detergent.

To achieve efficient solubilization of cellulose synthase TC from the membrane, five different types of detergents were included in the screening. After detergent treatment, the solubilized supernatant and unsolubilized pellet were separated by ultracentrifugation and subjected to Western blotting. All investigated detergents yielded similar amounts of solubilized AcsB, while only n-Dodecyl β-D-maltoside (DDM) productively solubilized AcsA (Fig 3A). These detergent solubilized membrane fractions were then used for subsequent purification using immobilized cobalt affinity chromatography (Co-NTA). AcsB, but not AcsC, was copurified by his-tagged AcsA. DDM solubilization treatment gave the highest yield with a nearly stoichiometric ratio for AcsA and AcsB. Other detergent treatments generated very low yields (Fig 3B).

**Fig 4:**
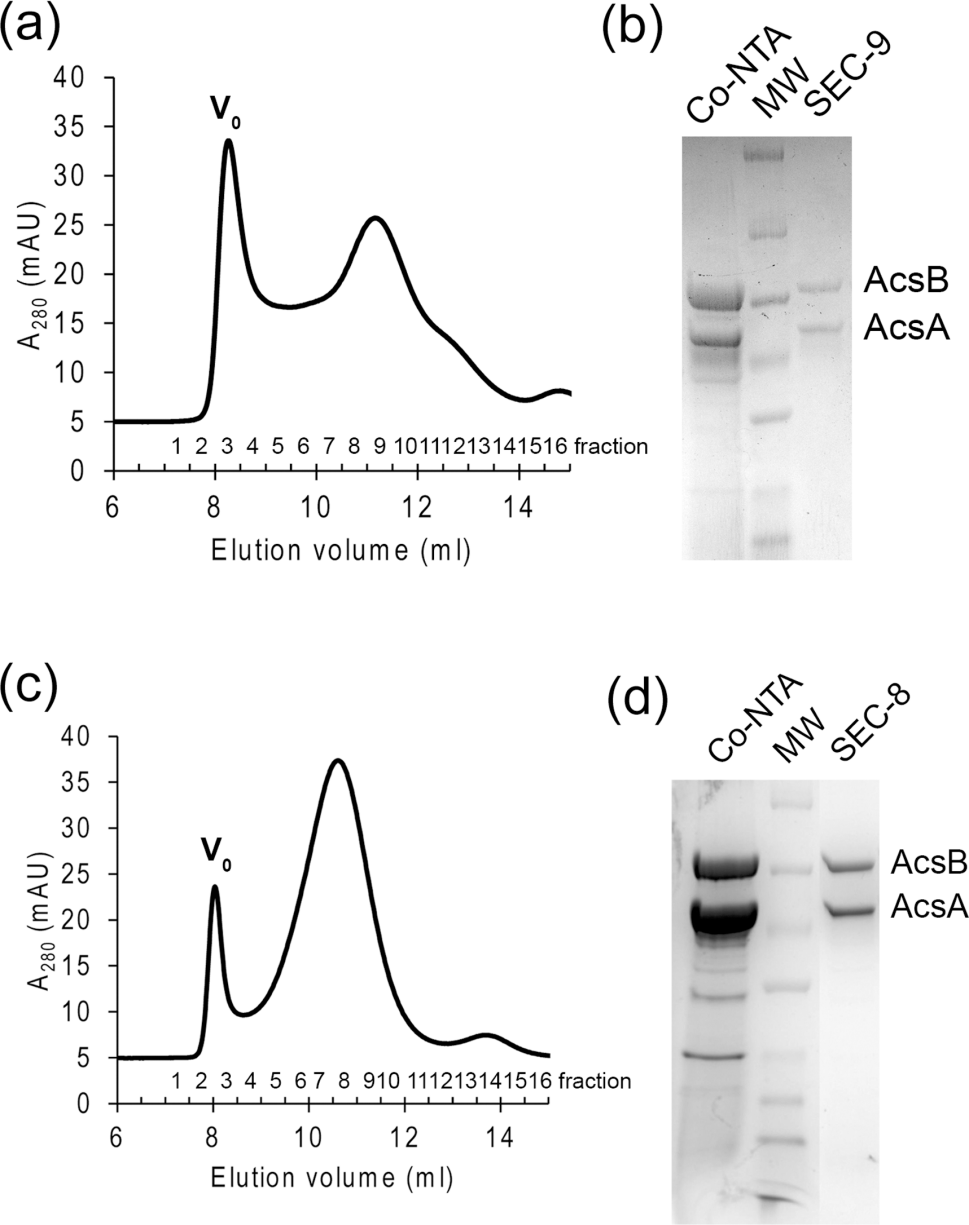
Detergent screening for the purification of metal chromatography and size exclusion chromatography. DDM solubilized supernatant was subjected to purification in buffer containing DDM (a and b) or LFCE14 (c and d). a and c. Size exclusion chromatography on the protein purified by Co-NTA resin. b and d. SDS-PAGE gels resolving the protein eluted from Co-NTA resin and major peak from size exclusion chromatography. The Co-NTA indicates elution from Co-NTA. The fraction 9 (SEC-9) of DDM purification and 8 (SEC-8) from LFCE14 purification were also analyzed in the gel. The bands of molecular weight in (b) from top to bottom are: 260, 140, 100, 70, 50, 40, 35 kDa. In (d), the bands of molecular weight from top to bottom are: 140, 100, 70, 50, 40, 35, 25, 15 and 10 kDa.

We also screened detergents for replacing DDM while the protein complex was on the Co-NTA resin in preparation for further purification by gel-filtration. Protein was equilibrated with buffer containing n-Dodecyl-N,N-dimethylamine-N-oxide (LDAO), n-Dodecyl-β-D-maltopyranoside (DDM), LysoFos® Choline Ether 14 (LFCE14) or Sodium taurocholate (NaTC), respectively, prior to elution with imidazole. As revealed by SDS-PAGE, both DDM and LFCE14 stabilized the complex during the affinity purification of his tagged protein (Fig 4B and 4D). With the presence of DDM in the buffer, the size exclusion chromatography displayed overlapping, multiple peaks (Fig 4A). In contrast, the protein purified in the presence of LFCE14 migrated in a major peak at ~11ml, which was shown to contain two distinct bands estimated to represent ~95 kDa and ~80 kDa polypeptides in SDS PAGE (Fig 4C and 4D). LDAO disassociated AcsA and AcsB and yielded non-stoichiometric amounts of the two proteins (S2A Fig). Using NaTC yielded AcsB, but no AcsA, possibly because this detergent made His-tagged AcsA precipitate on the Co-NTA column and AcsB dissociated and eluted alone (S2B Fig).

### Purified AcsAB Complex Is Able to Incorporate UDP-^14^C-Glucose

**Fig 5:**
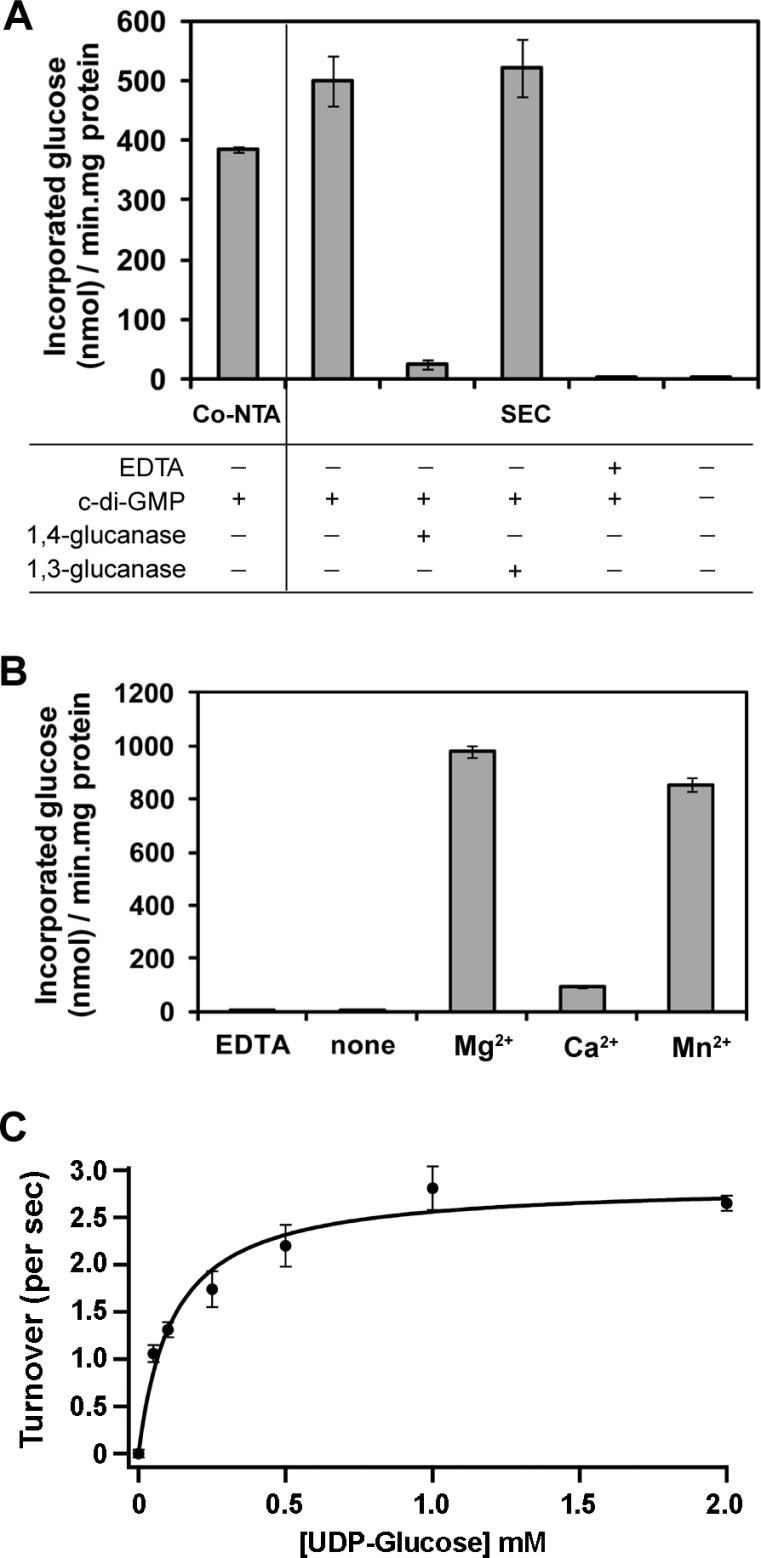
Detergent purified AcsAB complex showed *In vitro* cellulose synthase activity. (a) UDP-^14^C-glucose incorporation assay in Co-NTA (Co-NTA) or size exclusion chromatography (SEC) purified AcsAB. The purified protein was incubated with UDP-^14^C-glucose in the presence or absence of c-di-GMP and EDTA. The reaction products were subjected to β-1,4-glucanase (cellulase) and β-1,3-glucanase (callase) followed by scintillation counting to measure the remaining radioactive counts. (b) Requirement of cation for catalytic activity of AcsAB. Cation (20 mM) or EDTA (20 mM) was included in the reaction for 15 min which was then subjected to radioactivity measurement. (c) Michaelis-Menten kinetic assay of purified AcsAB, yielding constants K_M_ = 0.12 (± 0.03) mM UDP-glucose, and V_max_ = 1010 (± 60) nmol per mg protein complex per min.

To determine if the purified protein complex in detergent LFCE14 was functional for cellulose synthesis, the protein sample was used in a polymer incorporation assay in the presence of radioactive substrate UDP-^14^C-glucose [34, 42]. Radioactively labeled, water-insoluble product was quantified by scintillation counting. The results shown in Fig 5A suggest the purified protein was able to synthesize ^14^C labeled polymer when activator c-di-GMP and Mg^2+^ were included in the reaction buffer. The protein sample separated by size exclusion chromatography showed about 30% higher specific activity than the one directly eluted from Co-NTA resin, perhaps due to removal of non-functional protein aggregates and contaminant proteins by size exclusion chromatography (Fig 4D). The *in vitro* synthesized product was shown to be cellulose by digestion with β-1,4-glucanase (cellulase) and the fraction of insoluble product that can be attributed to callose was identified by digestion with β-1,3-glucanase (callase). The cellulase treatment solublized 95% ±1.5% of the radioactivity, while callase failed to release any radioactivity. When EDTA was used to remove cation, or when c-di-GMP was omitted, the protein was not able to catalyze label incorporation into cellulose (Fig 5A). Although we have been able to use transmission electron microscopy (TEM) to visualize *in vitro* synthesized cellulose fibrils from CesA prepared from the moss *Physcomitrella patens* [43], we saw no such fibers in the cellulose made from *G*. *hansenii* AcsAB, nor with cellulose synthesized by *R*. *sphaeroides* BcsAB [34].

Metal preference was determined by including various cations in the reaction. Mg^2+^ ion showed the highest enzyme activity, while Mn^2+^ was able to support almost as much activity. Addition of Ca^2+^ supported very low enzyme activity (Fig 5B). Monitoring activity in the presence of Mg^2+^ and at varied substrate concentrations showed that the enzyme has a KM of 0.12 (± 0.03) mM UDP-glucose and a Vmax of 1010 (± 60) nmol per mg protein complex per min for purified AcsAB. Assuming 100% of the protein was active, the enzyme turnover efficiency was 2.9 UDP-glucose molecules per AcsAB complex per second (Fig 5C).

### Low-Resolution 3D Reconstruction of AcsAB by Negative Stain EM

**Fig 6:**
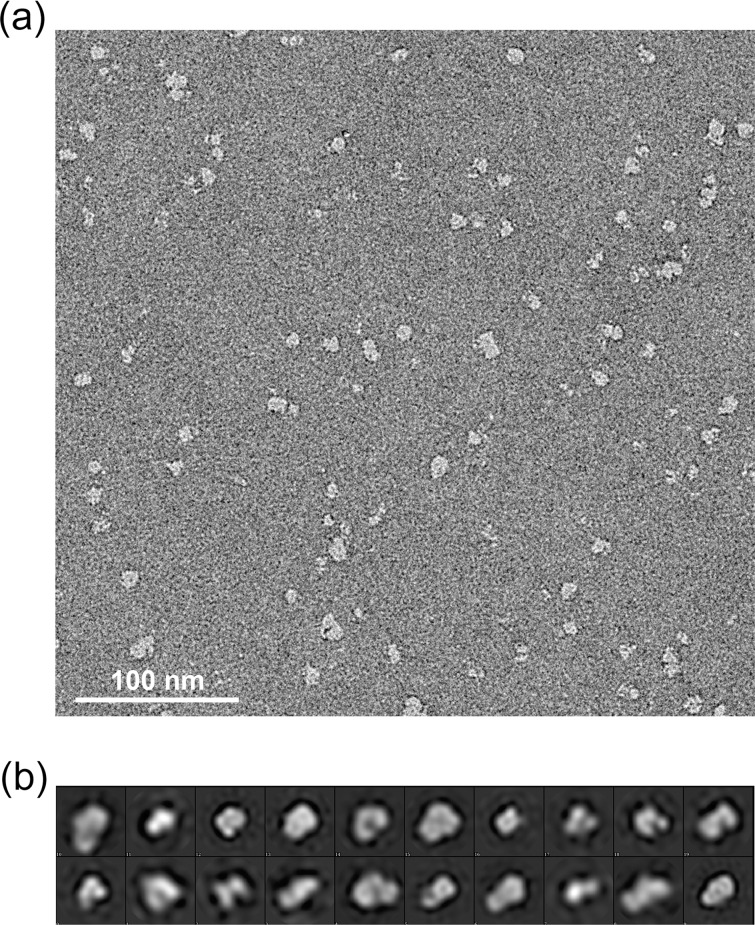
Electron microscopy of AcsAB purified by DDM indicates the presence of protein aggregates. (a) Representative electron micrographs of AcsAB purified in fraction 9 of size exclusion chromatography shown in Fig 4A. (b) 2D reference free class averages of negatively stained particles obtained in micrographs imaged from fraction 9.

AcsAB was 'polished' by size exclusion chromatography in buffers with different detergents as described above. We used negative stain EM to examine the material present in different peaks eluting from the size-exclusion columns developed with the various detergents. The products from the major peak (fraction 9 as labeled in Fig 4A) seen for DDM showed that this detergent promoted protein aggregation as also observed in the micrograph of negative stain EM (Fig 6A). Two-dimensional (2D) class averages for 3243 negatively stained particles further revealed the presence of a heterogeneous population of particles (Fig 6B).

**Fig 7:**
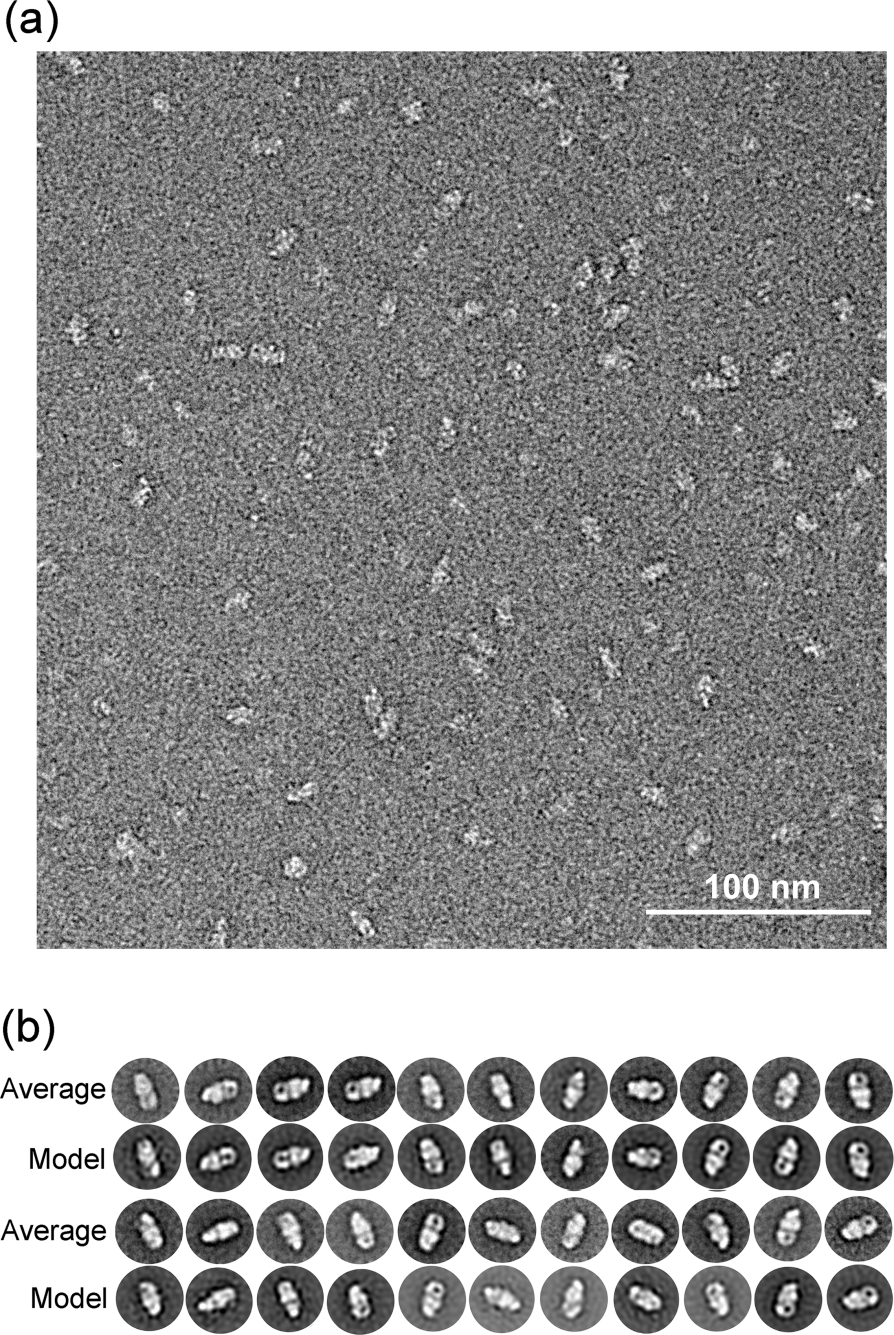
Visualization of LFCE14 purified AcsAB determined by negative stain EM. (a) Representative electron micrograph of LFCE14 purified AcsAB complex stained by uranyl formate. (b) 2D reference free class averages (odd-numbered columns) of negatively stained particles paired with projections of the final 3D model (even-numbered columns).

**Fig 8:**
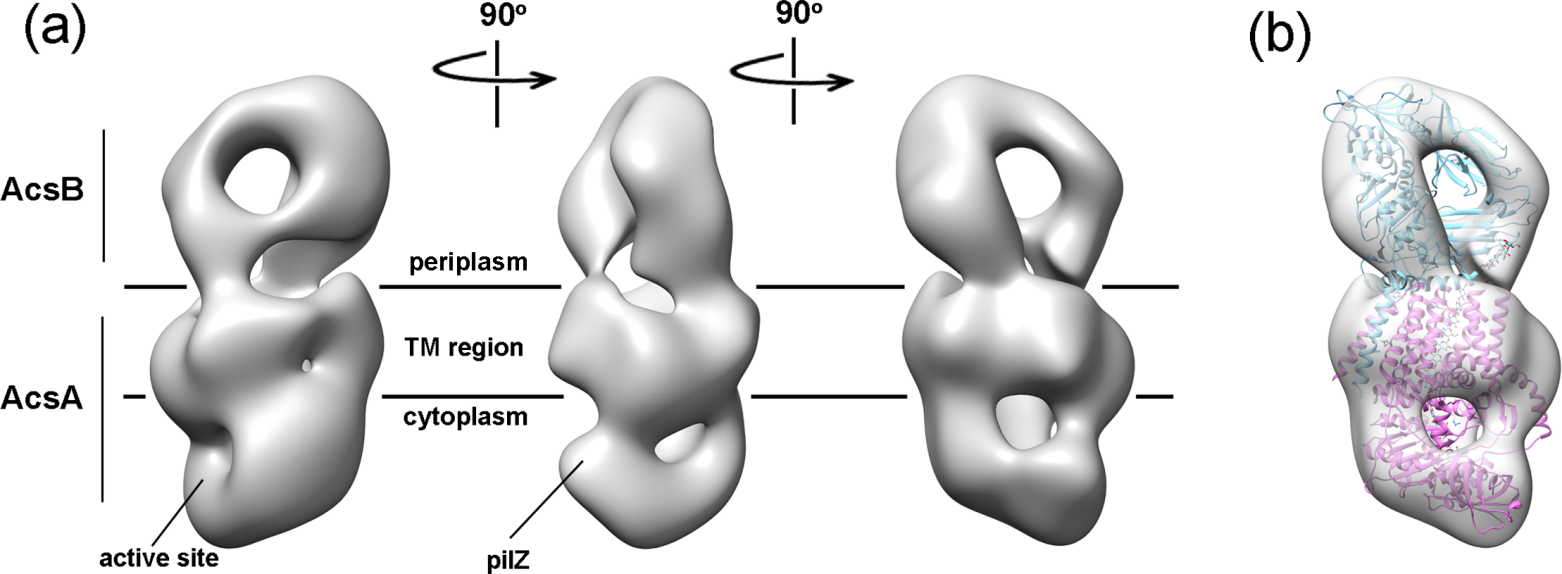
3D reconstruction of negatively stained AcsAB at 23.4 Å. (a) Three side views of 3D reconstructed model of AcsAB complex. The volume of AcsA is composed of membrane embedded TM region and large cytosolic region. The active site responsible for substrate binding and PilZ domain required for activator c-di-GMP binding are mapped in the cytosolic region. The density of AcsB sits on the top of AcsA. The cytoplasmic membrane boundaries are represented by black lines. (b) The AcsAB EM density map was docked with crystal structure of BcsAB (PDB entry 4HG6). BcsA and BcsB are shown in orchid and cyan ribbon representatives, respectively. The translocating glucan co-crystallized with BcsAB is indicated in cyan sphere.

**Fig 9:**
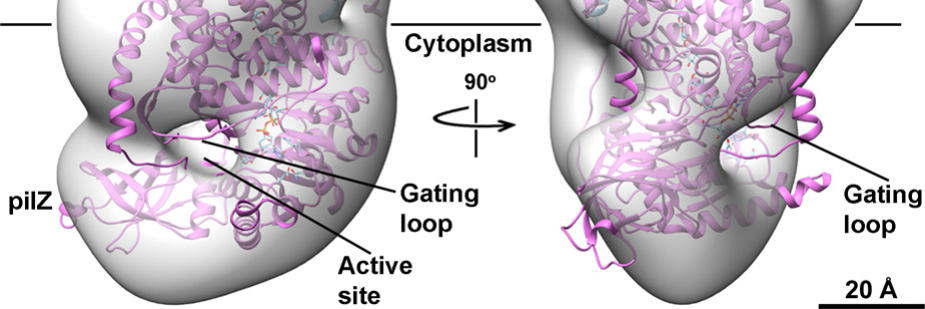
Architecture of of AcsA cytoplasmic domain. Surface representation of AcsA cytoplasmic domain revealed the active site as a pit to facilitate substrate UDP-glucose binding. The gating loop (IF3-TM7) identified in BcsAB crystal structure (PDB entries 4HG6 and 4P02) was not occupied in the AcsAB density map. The PilZ domain was located in the C-terminus of AcsA and represented by a protruding density. BcsA and translocating glucan are shown in orchid ribbon and cyan sphere representatives, respectively. The membrane boundary facing cytoplasm is indicated as a black line.

**Fig 10:**
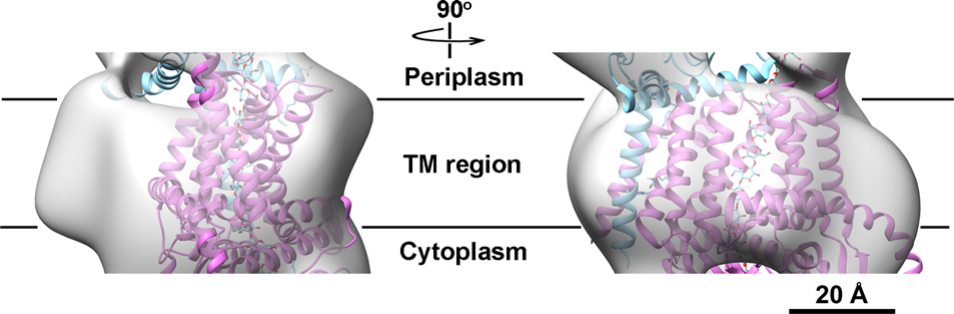
Larger TM region of AcsA compared with BcsA. Docking of the BcsA crystal structure into the AcsA EM density map revealed extra volume in the AcsA TM region rendered as a light gray surface. BcsA and BcsB are shown as orchid and cyan ribbons, respectively. The translocating glucan resolved in the BcsAB crystal structure is drawn as cyan spheres. The boundary of implied cytoplasmic membrane is indicated as a black line.

**Fig 11:**
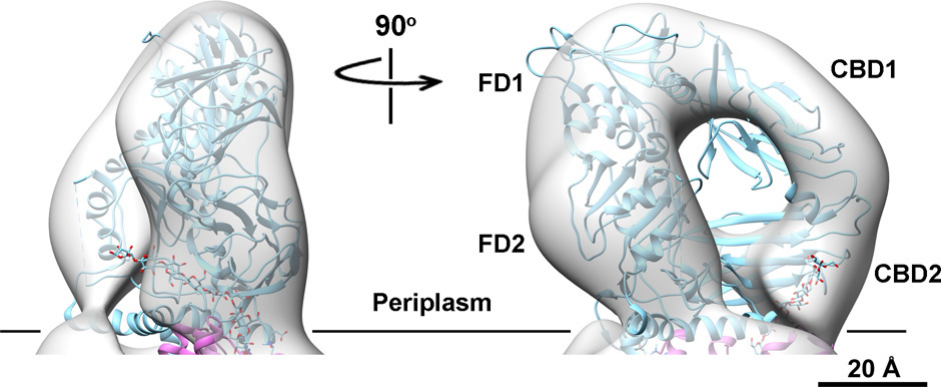
Domain organization of AcsB. The reconstructed 3D EM structure of AcsB fits well to the crystal structure of BcsB. Four conserved domains (FD1, FD2, CBD1 and CBD2) are defined in the density map. BcsB and translocating glucan are shown in cyan ribbon and sphere representatives, respectively. The membrane boundary facing periplasm is indicated as a black line.

The LFCE14 purified sample showed monodisperse particles on thin carbon layer coated grids. The negatively stained EM images revealed rod shaped particles in which one dimension was approximately half of the other dimension (particles averaging 150 Å in length and 70 Å in width; Fig 7A). Reference free 2D class averages were generated from a set of 3168 boxed particles and showed some details of the overall organization of the particles. Interestingly, a dark dot was visible in some of averages and projections, which suggested a cavity was present in the structure (Fig 7B). A three dimensional (3D) volume was reconstructed from the negatively stained particles at 23.4 Å resolution by using EMAN2 (Fig 8A, S3 Fig; deposition EMD-8075, wwPDB.org). The particles used for structure reconstruction covered most Euler angles (S4 Fig). Comparison of class averages (odd-numbered rows) and corresponding projections of the reconstructed 3D model (even-numbered rows) showed the final model to be internally consistent with the dataset of images (Fig 7B). The map was also validated by tilt-pair analysis [44, 45] in which computed orientation parameters clustered around the experimental tilt angle (10 degrees; S5 Fig). The crystal structure of BcsAB fit convincingly to the EM model (correlation coefficient of 0.84), indicating that the major features of AcsAB and BcsAB are conserved (Fig 8B). The alignment located AcsA, with its TM region embedded in the cytoplasmic membrane and extended cytoplasmic domain (Fig 8A and 8B). Segmentation analysis (SEGGER [46]) divided the AcsA volume into three sub-volumes that corresponded with the TM domain, the catalytic domain and the C-terminal domain (S6 Fig). The active site required for substrate binding was shown as a pit in the structure. The C terminal PilZ domain was revealed as a protrusion near the active site in the bottom of the 3D volume (Fig 9). As shown in Fig 10, the modeled volume occupying the TM region of AcsA was larger than that of the TM region of the BcsA crystal structure. The donut-shaped mass in the periplasm represented AcsB in close contact with the AcsA TM region. Segmentation analysis of the AcsB density revealed four sub-volumes, representing the predicted domains (FD1, FD2, CBD1 and CBD2) (S6 Fig). The observed cavity corresponding to the dark dot seen in some single particles and class averages was mapped in the center of AcsB, which is surrounded by subdomains resembling two cellulose binding modules and two ferrodoxin-like domains (CBD and FD, respectively, in Fig 11). Previous studies indicate that a gating loop (IF3-TM7) is located in front of the BcsA active site and controls the access to the active site by changing conformation [39]. The gating loop sequence is conserved in AcsAB (S7 Fig). The protein complex was purified in buffer lacking the activator c-di-GMP, presumably causing the gating loop to be in its 'resting' state blocking substrate access to the active site. However, as modeled the reconstructed AcsAB structure does not show volume for the gating loop (Fig 9).

## Discussion

**Fig 12:**
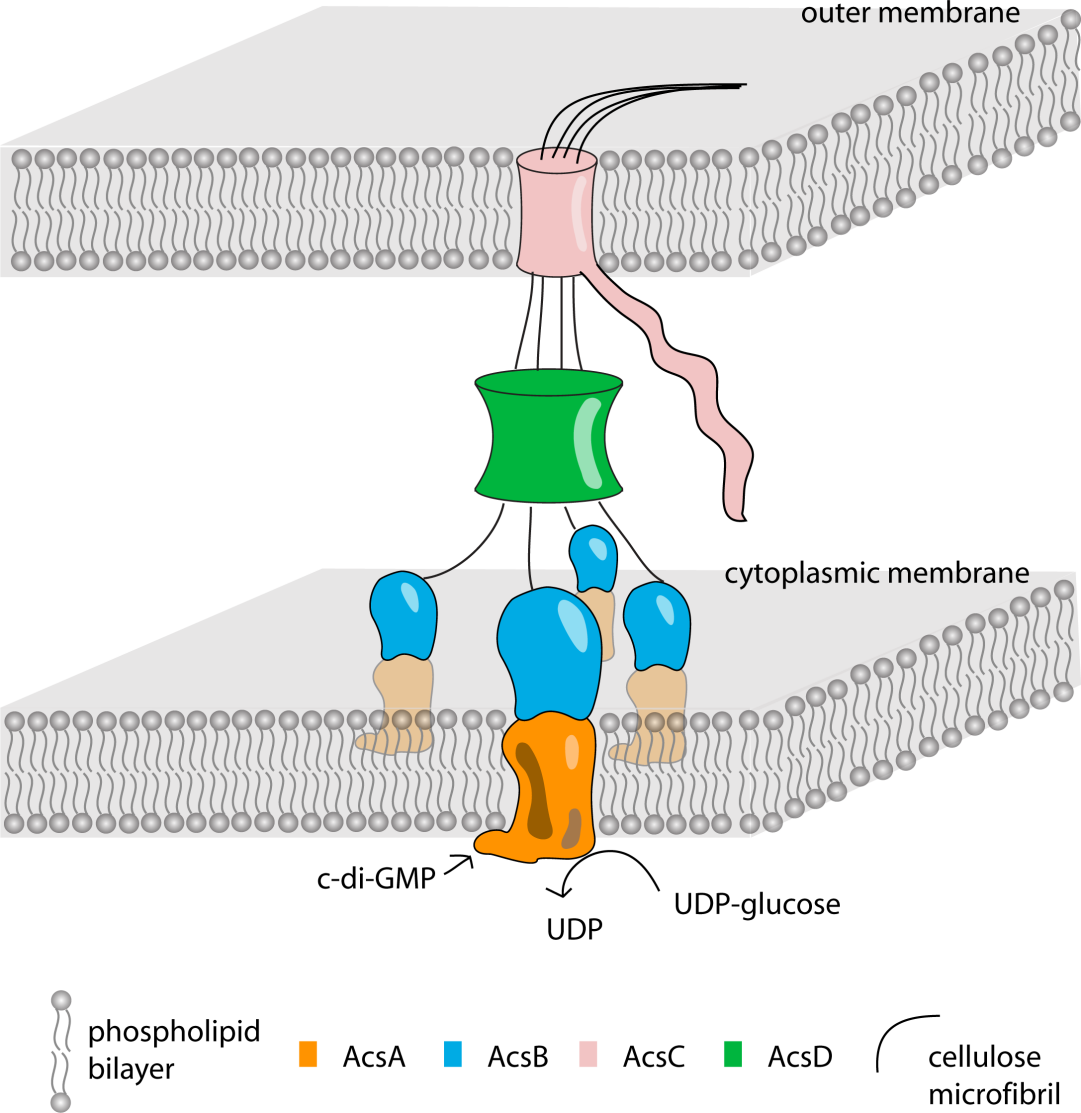
Schematic representation of proposed cellulose polymerization and secretion complex in *G*. *hansenii*. With the activation of c-di-GMP, cytoplasmic membrane embedded AcsAB complexes transfer glucose into synthesized glucan chain. The elongated glucan chains in close proximity are bundled and pass through the pathway made by AcsD and finally extruded out of cell through the AcsC channel on the outer membrane.

All bacteria that synthesize cellulose contain well conserved synthase genes. While most bacteria (like *R*. *sphaeroides*) appear to make amorphous cellulose, a few (like *G*. *hansenii*) make highly crystalline cellulose. The mechanisms that give rise to the different types of cellulose, intuitively involving linkage between synthesis and excretion from the cell, remain to be fully understood. For *G*. *hansenii*, this linkage must depend on structural features of the synthase (AcsAB) and accessory proteins (CcpAx, AcsD) required for translocation of cellulose across the periplasmic space and through the outer membrane (AcsC) (Fig 12).

Here we correct a frame shift in the 3'-end of the deposited sequence of *acsC*, GXY_04282 of ATCC 23769 [40], replacing the last 24 predicted residues and extending the protein by another 107 amino acids to fully define the β-barrel at the C-terminus. This protein is now predicted to be 1302 residues long, with mass of 138.6 kDa, in contrast to the previously predicted size of 1195 residues with 127.2 kDa mass. We note that the estimated size of AcsC from western blots is 138 kDa [41]. This size also agrees well with predictions from a second sequence deposited for the same *acs* locus of ATCC 23769 (GenBank AB091060.1). That sequence suggests that AcsC has the same number of residues and C-terminal extension as we report here. The two sequences are identical for all but 3 amino acid residues, presumably strain specific substitutions. Our observation that integrating a His-tag at the C-terminal end reported by Iyer et al. [40] prevented cellulose secretion by cultures of the mutant cell indicates that the full C-terminal end is needed to define the β-barrel pore for extrusion of cellulose. Deng et al. [41] reported that this region of sequence promoted transcription of *tetC* in *G*. *hansenii*, concluding that it encodes an independent promoter for transcription of *acsD*. We did observe that truncation of AcsC at the published C-terminal end had little to no effect on synthesis of AcsAB and AcsD, and the method of making the truncated AcsC left an intact copy of the *acsC* 3'-end immediately upstream of acsD. Perhaps the sequence performs a dual function, encoding the C-terminal end of AcsC and promoting transcription of *acsD*. Another possibility is that the inserted plasmid could have driven transcription of *acsD*. Expression of functional AcsC was not coupled to expression of the rest of the cellulose synthase machinery. There was also no apparent co-purification of any of those TC components to affinity purified, full length AcsC. It thus appears that AcsC interacts either transiently with the synthetic apparatus or indirectly, for example—via nascent cellulose emerging from AcsD. Alternatively, the associations may not have survived the detergents used to stabilize the membrane proteins.

We were able to optimize detergents and purification steps to prepare highly pure AcsAB that was catalytically active, cation-dependent, and properly regulated by c-di-GMP. The specific activity of AcsAB purified by product entrapment or metal affinity chromatography reported by McManus et al. [33] were one hundred fold lower than that reported for BcsAB [34]. The specific activity of AcsAB complex prepared with a final gel filtration chromatographic step (V_max_ of 1010 ± 60 nmol per mg protein complex per min) was about 30-fold lower than that of BcsAB, higher than that detected in the isolated cytoplastic membrane (52 nmol incorporated glucose/mg.min), the material obtained by one step product entrapment (420 nmol incorporated glucose/mg.min) and the metal affinity purified AcsAB (660 nmol incorporated glucose/min.mg) [25, 26, 33]. Assuming all protein molecules in our sample are functional and contributing to the detected activity, the turnover number of AcsAB is about 2.9 molecules per protein complex per sec, which is 1.7-fold higher than that detected in affinity purified AcsAB (1.72 ± 0.06 molecules per protein complex per sec) [33]. However, it is about 30 times lower than the efficiency of BcsAB (90 molecules per protein complex per sec) [34].

The yield of purified AcsAB complex was sufficient to determine a negative stain EM structure at the resolution of 23.4 Å. At that resolution, we were able to ask if the structure of AcsAB synthase differs from BcsAB synthase, finding that overall they are similar. This includes c-di-GMP binding domain, substrate binding cleft, and juxtaposition between the A and B components. The membrane spanning region of AcsAB did appear larger than for BcsAB, but there are no amino acid insertions in AcsA (S7 Fig) or AcsB [34] that could explain that difference. Considering the extra density was specifically observed in the TM domain, but not in periplasmic and cytosolic domains, we suggest the extra density reflects the detergent micelle surrounding the TM domain. Such micelles have been seen in EM maps representing TM domains of other detergent purified membrane proteins [47–49]. The AcsAB structure did not contain volume for the gating loop conserved with BcsA (FxVTxK motif, between IF3 and TM7 in S7 Fig), which may reflect the low resolution of the structure or suggest movement of the loop. We tentatively conclude that *G*. *hansenii's* ability to make crystalline cellulose does not depend on differences in synthase structure from that of BcsAB; rather, it depends upon different organization of the AcsAB synthase molecules within the cell.

Four schemes exist for how bacterial cells organize their genes devoted to cellulose synthesis (reviewed in [35]). The process of cellulose synthesis includes polymerization, assembling and secretion of glucan chains, which in *G*. *hansenii* requires the function of other associated proteins such as β-1,4-glucanase CMCax, periplasmic proteins AcsD and CcpAx, and pore-forming protein AcsC [19, 21, 22, 33]. A previous study was able to see in vitro synthesized cellulose associated with doughnut-shaped particles, which were 9–18 nm in thickness and 22–55 nm in diameter [25]. These particles are larger than the size expected for the monomeric complex of 83kDa (AcsA) and 93kDa (AcsB) [25]. This is verified by the negative stain TEM structure we present for AcsAB. It is possible that the higher ordered oligomers needed to make cellulose fibrils or ribbons were disrupted during the detergent-based purification; alternatively, the associations between AcsAB and other necessary components may be weak and transient in nature, and maintained by the presence of cellulose itself. In this regard, cellulose biosynthesis machinery appears different from the machines that make extracellular EPS and capsular CPS, where a fixed Wca-Wcz complex or ABC transporter mediates translocation from inner membrane across the periplasm to the exit portal in the outer membrane [50, 51]. We favor the model presented in Fig 12 for the *G*. *hansenii* apparatus.

The model must account for the following observations. First, AcsAB is of known size, and occupies the inner membrane and partial periplasm—the rest of the periplasm and outer membrane must be bridged by other components. Second, the crystal structure of AcsD revealed a cylinder shaped homo-octamer with ~65 Å in height, ~90 Å in outer diameter and ~25 Å in inner diameter. The octamer presents four inner passageways for the extrusion of individual glucan chains [36]. Third, domain predictions suggest that AcsC has seventeen TPR-like motifs in the N-terminus and a β-barrel porin at the C-terminus (S1 Fig and S8 Fig). The barrel is made of 14, membrane spanning β-strands that form an oval shaped channel. The inner diameter of the predicted channel is ~17 Å x 29 Å on its short and long axes and has the capacity for ~4 glucan chains (S8 Fig). The TPR-motifs in AcsC can be compared to periplasm located proteins such as AlgK known to be active in other sugar polymer secretion systems. Like AcsC, AlgK contains repetitive TPR motifs that, in the case of AlgK, mediate protein-protein interaction [52–55]. AlgK is essential for the proper localization of porin AlgE and interpreted to serve as a scaffold to link the inner and outer membrane portion of the complex [56, 57]. From such a comparison, it is likely that AcsC contains a fusion of such functions, which implies a crucial role of the periplasmic portion is to facilitate transportation of glucan to and through the porin [57]. AcsC may interact with periplasmic proteins (eg. AcsB, AcsD, cellulase) and guide the nascent glucan in entering the porin. The co-crystallization of BcsAB along with a translocating glucan indicated that one nascent glucan chain is polymerized by one catalytic subunit [38], but not two [36]. Taken together, we propose a model of cellulose synthase complex in Gluconacetobacter (Fig 12), in which tetramers of AcsAB in the inner membrane cooperate with the partners located in periplasm and outer membrane to synthesize and deliver growing glucan chains that further assemble into elementary fibrils, and from there into ribbons.

Higher resolution information coupled with an understanding of interactions with other TC components is required to determine the basis for that organization. Although cryoEM has traditionally been appropriate for structural studies of proteins ≥200 kDa in mass, recent advent of direct electron detectors may make it suitable for smaller ones like the ~180 kDa AcsAB complex [58–61]. Such detectors have also dramatically improved attainable resolution, with electron density maps defined at ~3 Å being frequently reported [59, 61–66]. DDM was determined as the best detergent for protein solubilization from membrane, but final elution from affinity columns and storage of the AcsAB complex in buffer containing LFCE14 was required to obtain aggregate free material. Such samples could be used to explore feasibility of cryoEM.

## Materials and Methods

### Construction of the Strain Used to Express Cellulose Synthase Complex

Homologous recombination was used to generate a *G*. *hansenii* strain with His-tag at the C terminus of AcsC. The C terminal sequence of AcsC-T and AcsC-C fused with 12 histidine encoding sequence was amplified using PCR primers AcsC-T-HR and AcsC-C-HR (S1 Table), respectively. The DNA sequence encoding tetracycline resistance gene was amplified from the plasmid pUCD2 by using the primer Tet (S1 Table). The fragments of AcsC and Tet were integrated into the plasmid pUC19 linearized by restriction enzyme BamHΙ and EcoRΙ (New England Biolabs) via In-Fusion cloning kit (Clontech) to generate pUC19AcsC-T-His and pUC19AcsC-C-His, which were transformed into the wild type strain by electroporation. The transformants were selected against 10 μg/ml tetracycline in the culture medium.

In our second strategy to express CSC, we chose to clone the entire set of *acs* genes onto a plasmid that could be maintained in *G*. *hansenii* itself. This approach assured that correct post-translational processing would occur to split AcsAB into separate AcsA and AcsB polypeptides. The promoter of the *acs* operon and coding sequences of AcsC and AcsD were amplified using the primer of acs promoter-1 and AcsCD (S1 Table), respectively. The plasmid pUCD2 was linearized by PvuΙ and SalΙ. Then the promoter and AcsCD were joined to the linearized plasmid using the In-Fusion cloning kit to generate pUCD2P_acs_::CD. The acs operon promoter and His12-AcsAB were amplified by using primer acs promoter-2 and HisAcsAB (S1 Table), respectively. Then these two fragments were integrated into linearized pUCD2P_acs_::CD by enzyme SalΙ to produce pUCD2CDHisAB, which was transformed by electroporation into wild type strain and plated on the agar plates containing 100 μg/ml spectinomycin.

To assay for cellulose production, the colonies plated on SH agar plates were inoculated into Schramm-Hestrin (SH) media containing proper antibiotics and incubated at 30°C without shaking for 3 days. Cultures producing cellulose (Cel^+^) were able to produce pellicle at the media surface, while those not producing cellulose (Cel^-^) failed to make pellicle.

### SDS-PAGE and Western Blot

SDS-PAGE was performed using a well established method [67]. *Gluconacetobacter* cells were disrupted using a micro-sonication probe for three pulses at duration of 10 sec and interval of 3 min in ice-water bath (Artek Sonic Dismembrator Model 150) that was followed by the determination of protein concentration by the Bradford method [68]. The same amount of protein (10 μg) for each sample harvested at the indicated OD value was incubated with loading buffer for 10 min at 65°C and resolved with 4–12% gradient SDS-PAGE gel (GenScript) in Tris-MOPS buffer. To monitor different fractions during solubilization optimization, the same volume of sample (20 μl) was separated by SDS-PAGE. Proteins were then transferred onto nitrocellulose membranes in a BioRad Mini-Transfer Cell for 60 min. The membrane was blocked by incubating with 5% BSA for 30 min before adding primary, polyclonal antibodies against AcsA, AcsB or AcsC, respectively. (The antibodies, generously provided by Ming Tien, were raised from peptide fragments and then purified by binding to and eluting from peptide on nitrocellulose membrane.) After washing blots in Tris Buffered Saline with Tween® 20 (TBST) buffer, Anti-Rabbit IgG (whole molecular)-Alkaline Phosphatase (Sigma) was diluted 1:6,666 and incubated with the membrane for 30 min. Blots were then thoroughly washed and incubated with 5-bromo-4-chloro-3'-indolyphosphate p-toluidine salt (BCIP)/nitro-blue tetrazolium chloride (NBT) solution (Amresco) for immuno-staining of blotted protein. Alternatively, the Cobalt (Co)-NTA resin purified protein (10 μl) was directly load on SDS-PAGE for coomassie staining.

### Purification of AcsAB Complex and AcsC Protein

The engineered strain was plated onto SH agar plates with 100 μg/ml spectinomycin and incubated at 30°C for 3 days. All of the cells on the agar plate were resuspended and inoculated in liquid SH media containing cellulase (0.2 g/liter) to produce cultures free of accumulating cellulose [25]. The resulting cultures were harvested at the indicated OD value after incubation with shaking at 30°C. Then the cells were resuspended in ice-cold lysis buffer (20 mM NaH_2_PO_4_ pH7.2, 300 mM NaCl and 5 mM cellobiose) and lysed in a microfluidizer at 20,000 psi. The membrane fraction was pelleted by centrifugation in a Beckman Ti45 rotor at 120,000 x g for 60 min at 4°C and solubilized at 4°C in buffer (SB) containing 20 mM NaH_2_PO_4_ pH7.2, 300 mM NaCl, 5 mM cellobiose, 5 mM MgCl_2_, 10% glycerol, 20 mM imidazole and specified detergent. To screen for solubilization, 1% LDAO, 1.5% DDM, 2% Polyoxyethylene(8)dodecyl ether (C12E8), 2% Triton and 3.2% NaTC were gently mixed with pelleted membrane fraction at 4°C for 60 min. The solubilized supernatant was collected by centrifugation at 4°C in a Beckman Ti45 rotor at 120,000 x g for 45 min and incubated with Co-NTA resin (Thermo scientific) for 60 min with gentle agitation at 4°C. Subsequently, the resin was packed in a gravity flow column and washed with 10 column volumes of SB buffer containing 30 mM imidazole and indicated detergent. Then elution buffer (20 mM Tris-HCl pH7.5, 100 mM NaCl, 5 mM Mg^2+^, 5 mM cellobiose, 10% glycerol, 300 mM imidazole and indicated detergent) was used to elute protein from the resin. The eluate was concentrated to about 500 μl using a centrifugal concentrator (100K cutoff, Pall) and applied to a Superdex 200 gel filtration column (GE healthcare, Superdex^TM^ 200 10/300GL, 24ml).

### Cellulose Synthase Assay in Detergent-Purified AcsAB Complex

Cellulose synthase activity was determined by measuring the incorporation of UDP-^14^C-glucose (Perkin Elmer) into insoluble glucan chains. The reaction was performed in buffer containing 20 mM Tris-HCl pH7.5, 100 mM NaCl, 5 mM cellobiose, 20 mM MgCl_2_, 30 μM c-di-GMP, 2 mM UDP-glucose and 2 μM UDP-^14^C-glucose. After adding 32 μg (for Co-NTA elution) or 7.3 μg (for gel filtration) of the protein sample, reactions were immediately placed into a 30°C water bath and incubated for 30 min. Then 2% SDS was added to terminate the reaction. To precipitate the synthesized product, the reaction was centrifuged at 16,000 x g for 10 min at room temperature. The pellet was then washed once at room temperature with 50 mM NaAc (pH5.5) and resuspended in the same buffer. For cellulase and callase digestion, 10 units per reaction of cellulase (Worthington) or 5 units per reaction of callase (Megazyme) were incubated with the synthesized products overnight at 50°C and 37°C, respectively. Finally, all of the reaction solution was filtered through a glass fiber filter (Whatman, GF/C, diameter 24mm) and washed with 4 ml 66% ethanol, 4 ml water and 4 ml ethanol in sequencial. After drying, the glass filter was submerged in a scintillation cocktail (ScintiVerse™ BD, Fisher) for counting radioactive decay.

To identify metal preference, the same procedure was followed as above except the reaction time was 15 min instead of 30 min and 20 mM concentration of the indicated metals was present. The enzyme kinetics assay was performed in the presence of varying amount of substrate. All the measurements were performed in three replicates and the value is the mean of three replicates ± standard deviation. Michaelis-Menten analysis was performed using non-linear regression, fitting the above data to the standard equation v = V_max_*[S]/(K_M_ + [S]) with a program created using Lahey Fortran F77L (Lahey Computer Systems, Inc., Incline Village, NV) and NONLIN (Michael Johnson, University of Virginia, Charlottesville, VA).

### Negative Staining Sample Preparation and Imaging

Three and one half μl of protein sample at a concentration of 0.002 mg/ml was adsorbed on glow discharged grids (Pelco easiGlow) coated with thin layer of continuous carbon film deposited under high vacuum (10^−6^ Torr, Denton Vacuum model DV-502B). Grids were then washed and stained in 10 drops of 1% (wt/vol) of freshly prepared uranyl formate and allowed to air dry. Image acquisition of negative stained grids was performed in a Tecnai T12 transmission electron microscope operating at 120 keV using a dose of ~20 e^-^/ Å^2^ and phase contrast was optimized by defocusing over a range of 0.5–2.5 μm. Images were obtained using a 4k x 4k charge-coupled device (CCD) camera (Eagle, FEI) with a magnification of 68,000 x (1.45 Å/pixel on the specimen).

### EM Data Processing

Image evaluation and particle picking were performed using the software EMAN2 [69]. The contrast transfer function (CTF) parameter of each micrograph was determined with CTFFIND3 [70]. In total 3168 particles were manually picked and extracted in 208 pixel x 208 pixel boxes. Boxed particles were normalized and subjected to reference-free 2D class averaging in ISAC [71], which extracted 'stable classes' by repeated cycles of clustering to assess reproducibility of class assignments. With no symmetry imposed (sym = C1), an initial 3D model was generated from 2D classes using the program of e2initialmodel.py in EMAN2. The initial model was used as a start model to refine the final 3D reconstruction by using the program of e2refine_easy.py in EMAN2. The refined model was deposited to the EMDataBank as EMD-8075 (wwPDB.org). To obtain a molecular interpretation of AcsAB density map, a rigid-body docking of BcsAB atomic model into AcsAB EM map was performed by using the function 'Fit in Map' in UCSF-Chimera [72]. The AcsAB showed 84% of correlation with the simulated BcsAB model at 24 Å. The function 'SEGGER' [46] in Chimera was used for segmenting the 3D density map.

### Tilt-Pair Validation

Negatively stained grids were prepared as described above. In the same specimen area, untilted and 10° tilted images were sequentially acquired at a dose rate of ~20 e^-^/ Å^2^. Processing, particle-picking and tilt validation were performed using EMAN2. In total 120 particle pairs were extracted from 7 pairs of CTF-corrected images.

## Supporting Information

S1 Fig Sequence comparison and domain organization of truncated and complete AcsC of *G*. *hansenii* ATCC 23769. The amino acid sequence of truncated (AcsC-T) and complete (AcsC-C) AcsC are aligned, with the location of the frame shift denoted by switching from black to gray AA symbols. Predicted TPR repeats and β–barrel are shown by gray shading and black boxes, respectively.
(PDF)

S2 Fig Dissociation of AcsAB complex by detergents LDAO and NaTC. (a) Size exclusion chromatography and staining an SDS-PAGE gel with Coomassie Blue shows disassociation of AcsA and AcsB during purification in the presence of LDAO. (b) Purification using buffer containing NaTC caused His-tagged AcsA to remain on the Co-NTA resin. In the SDS-PAGE gel, "Co-NTA" represents the protein eluted from the Co-NTA resin and the numbers indicate the fraction separated by size exclusion chromatography.
(PDF)

S3 Fig Gold-standard Fourier shell correlation (FSC) plot of the final 3D refinement. The density map is resolved to 23.4 Å according to the FSC value of 0.143.
(PDF)

S4 Fig Orientation distribution of all particles used for final 3D reconstruction. Cylinders of varying height represent the number of particles found in given orientations. Higher and lower cylinders are in light grey and black, respectively.
(PDF)

S5 Fig Tilt-pair validation of the AcsAB density map. Sixty-eight percent of effective particle pairs cluster around the experimental tilt angle of 10 degrees, though some spread indicates uncertainty in determining particle orientations that is typical of smaller structures. Each black dot represents the tilt axis and tilt angle for a particle pair in polar coordinates. The red circle is centered at the expected relative tilt angle 10°. The outer radius of the plot is 180°.
(PDF)

S6 Fig Segmentation of the reconstructed 3D density map. AcsA was segmented into three sub-volumes corresponding to its TM domain (TMD), catalytic domain and C-terminal domain in light blue, light green and springgreen, respectively. AcsB was segmented into four regions: FD1 in yellow, FD2 in violet, CBD1 in orange and CBD2 in purple.
(PDF)

S7 Fig Sequence comparison and domain organization of *G*. *hansenii* AcsA and *R*. *sphaeroides* BcsA. The fused AcsAB sequence is separated into AcsA and AcsB based on previous reports [24, 37]. Secondary structure elements are shown with their corresponding primary amino acid sequences. The major structural domains of BcsA revealed by published crystal structure are shown above the alignment. The conserved transmembrane helices (TMHs), interfacial (IF) helices, and PilZ-domain are indicated with dark grey shade, light grey shade and black box, respectively.
(PDF)

S8 Fig Structure prediction of AcsC by ITASSER. According to the domain prediction of AcsC as shown in S1 Fig, the amino acid sequence of AcsC was split into N-terminal and C-terminal portions. After removing the signal peptide, the N-terminal portion (33-942aa.) and C-terminal portion (943-1302aa.) were separately subjected to structure prediction using ITASSER. (a) Prediction for the N-terminal portion (C-score = 0.53, Estimated TM-score = 0.78±0.09, Estimated RMSD = 7.5±4.3Å). (b) and **(c)** Prediction for the C-terminal portion (C-score = -3.18) as shown in side view (b) and end view (c), reproduced to the right with 4 strands of cellulose (Iβ crystalline form) inserted into the channel.
(PDF)

S1 Table PCR primers used in this study. (PDF)
