# Supplementary material for: Structure of the Cellulose Synthase Complex of Gluconacetobacter hansenii at 23.4 Å Resolution
Source: PLoS One. 2016 May 23;11(5):e0155886. doi: 10.1371/journal.pone.0155886 (PMC4877109; doi:10.1371/journal.pone.0155886)
Supplement: S1 Fig — The amino acid sequence of truncated (AcsC-T) and complete (AcsC-C) AcsC are aligned, with the location of the frame shift denoted by switching from black to gray AA symbols. Predicted TPR repeats and β–barrel are shown by gray shading and black boxes, respectively. (PDF) [file pone.0155886.s001.pdf]

S1 Figure

```
AcsC-C  MTHKRYASSLSAGLLATTCVAGLLLQANGARAQQAAEAQAPASSTTMMQAATVAPAQSGQAAVVQRLVQQARFWMQQHQYENARQSLQSAARLAPDSVDL  100
AcsC-T  MTHKRYASSLSAGLLATTCVAGLLLQANGARAQQAAEAQAPASSTTMMQAATVAPAQSGQAAVVQRLVQQARFWMQQHQYENARQSLQSAARLAPDSVDL  100

AcsC-C  LEAEGEYQSHIGNRDAALDTQRRLHQAAPGSTYESQLNDLLHEQAISQPDLAHARSLAASGHSDQAVEAYQHLFNGSTPTPSLAVEYYQTLAGVSGQAGT  200
AcsC-T  LEAEGEYQSHIGNRDAALDTQRRLHQAAPGSTYESQLNDLLHEQAISQPDLAHARSLAASGHSDQAVEAYQHLFNGSTPTPSLAVEYYQTLAGVSGQAGT  200

AcsC-C  AQDGLIRLVKANPSDFRAQLALAQVLTYQPGTRMEGLQRLQALQKYQSSAPVEAATAEKSYRQTLSWLPVTPETLPLMQKWLDAHPSDSALRTHMAEPAG  300
AcsC-T  AQDGLIRLVKANPSDFRAQLALAQVLTYQPGTRMEGLQRLQALQKYQSSAPVEAATAEKSYRQTLSWLPVTPETLPLMQKWLDAHPSDSALRTHMAEPAG  300

AcsC-C  GPPDKGALARQDGFKALNAGRLSAAQAAFQSALNLNAKDGDALGGLGLVAMRAGHNEEAHRYLEDAIAADPKNAAHWRPALAGMAVGEEYGSVRRLIASG  400
AcsC-T  GPPDKGALARQDGFKALNAGRLSAAQAAFQSALNLNAKDGDALGGLGLVAMRAGHNEEAHRYLEDAIAADPKNAAHWRPALAGMAVGEEYGSVRRLIASG  400

AcsC-C  QTQEAEQRLMTLARQPGQSEGATLMLADLQRSTGQTGEAERNYRAILARNGDNPIALMGLARVLMGEGQENEANALLSRLGGRYSDQVQQIEVSGIMAEA  500
AcsC-T  QTQEAEQRLMTLARQPGQSEGATLMLADLQRSTGQTGEAERNYRAILARNGDNPIALMGLARVLMGEGQENEANALLSRLGGRYSDQVQQIEVSGIMAEA  500

AcsC-C  ARTSDSAQKVSLLRQAMTKAPDDPWLRINLANALQQQGDSAEAANVMRPLLTSPRTPADYQAAILYASGNGNDTLARRLLAGLSPDDYSPAIRTIADEMA  600
AcsC-T  ARTSDSAQKVSLLRQAMTKAPDDPWLRINLANALQQQGDSAEAANVMRPLLTSPRTPADYQAAILYASGNGNDTLARRLLAGLSPDDYSPAIRTIADEMA  600

AcsC-C  IKADLASRLSMVSNPTPLVREALAAPDPTGARGVAVADLFRQRGDMLHAHMALRIASTRNIDLTTEQRLAYATEYMKISNPVAAARLLAPLGDGSGTATG  700
AcsC-T  IKADLASRLSMVSNPTPLVREALAAPDPTGARGVAVADLFRQRGDMLHAHMALRIASTRNIDLTTEQRLAYATEYMKISNPVAAARLLAPLGDGSGTATG  700

AcsC-C  SAMSPDQRQTLMQLRMGISVAQSDLLNQRGDQAAAYDHLAPALQADPEATSPKLALARLYNGRGKYGHALDIDLAVLRHNPQDLDARQAAVQAAANDGKD  800
AcsC-T  SAMSPDQRQTLMQLRMGISVAQSDLLNQRGDQAAAYDHLAPALQADPEATSPKLALARLYNGRGKYGHALDIDLAVLRHNPQDLDARQAAVQAAANDGKD  800

AcsC-C  NLAMQLAQDGVQQSPMDARSWLGMAVADRAVGHGDRTLADLRRAYELRLQQLKISRGDAIGGDETQATAPPTANPFRRDAYGHALSLGAPPGENGYSTAG  900
AcsC-T  NLAMQLAQDGVQQSPMDARSWLGMAVADRAVGHGDRTLADLRRAYELRLQQLKISRGDAIGGDETQATAPPTANPFRRDAYGHALSLGAPPGENGYSTAG  900

AcsC-C  SVPEISDQMLSSINGQIHTLSEDMAPSVDAGLGFRVRSGTPGMGALTEASVPIVGRIPLQAGTSALTFTATPTFLTSGHLPQTGYDIPRFGTNLFALERN  1000
AcsC-T  SVPEISDQMLSSINGQIHTLSEDMAPSVDAGLGFRVRSGTPGMGALTEASVPIVGRIPLQAGTSALTFTATPTFLTSGHLPQTGYDIPRFGTNLFALERN  1000

AcsC-C  LQNQNNSAEHRINTDTIGREAGVAPDVRFANNWVSADVGASPLGFTLPNVIGGVEFAPRVGPVTFRVSGERRSITNSVLSYGGMTDALTGKKWGGVVTNH  1100
AcsC-T  LQNQNNSAEHRINTDTIGREAGVAPDVRFANNWVSADVGASPLGFTLPNVIGGVEFAPRVGPVTFRVSGERRSITNSVLSYGGMTDALTGKKWGGVVTNH  1100

AcsC-C  FHGQVEATLGNTIVYGGGGYAIQTGHHVQSNTEVEGGLGANTLVYRNRKHEVRVGVNLTYFGYKHNEDFYTYGQGGYFSPQSYFAATVPVRYSGHSGLFD  1200
AcsC-T  FHGQVEATLGNTIVYGGGGYAIQTGHHVQSNTEVEGGLGANTLVYRNRKHEVRVGVNLTYFGYKHNEDFIYLRAGWLLLAAVLFCGN------GARPVFR  1194

AcsC-C  WDVTGSIGYQLFHEHSSAFFPTNPVYQALANGLAGVSTAELSLESARYPGDDVGSLVGGFDGRVGYRVSHSLRLDLSGRFQKAGNWDEGGAMISAHYLIM  1300
AcsC-T  A---------------------------------------------------------------------------------------------------  1195

AcsC-C  DQ                                                                                                    1302
AcsC-T  --                                                                                                    1195
```
