# Supplementary figures and images for: Structure of the Cellulose Synthase Complex of Gluconacetobacter hansenii at 23.4 Å Resolution
Source: PLoS One. 2016 May 23;11(5):e0155886. doi: 10.1371/journal.pone.0155886 (PMC4877109; doi:10.1371/journal.pone.0155886)

S2 Figure

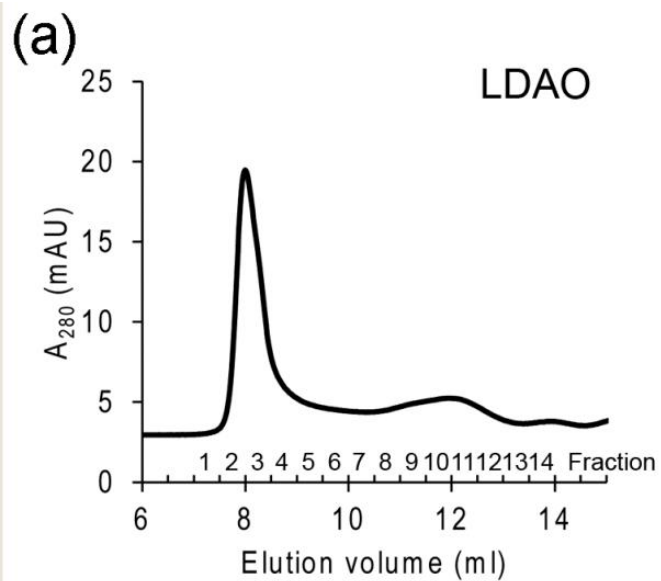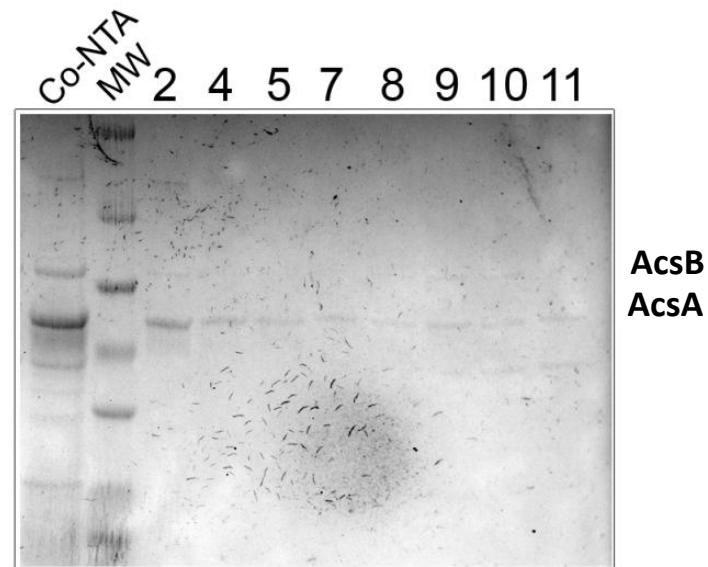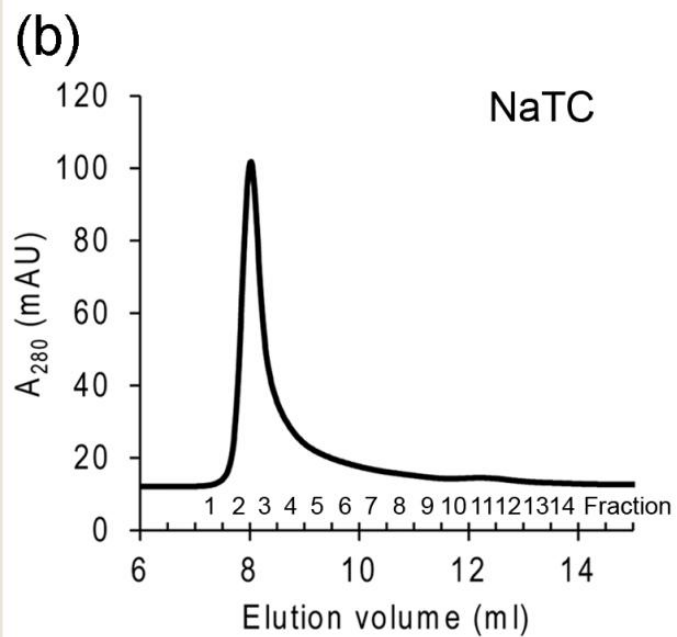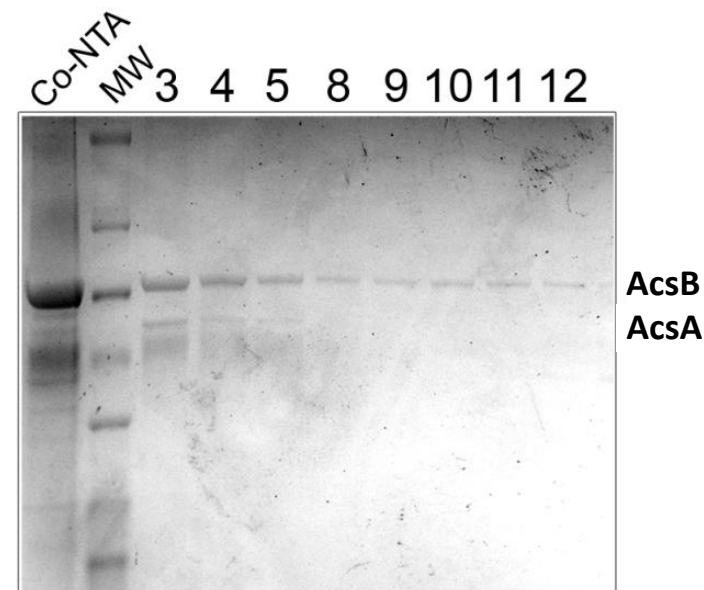

Supplement: S2 Fig — (a) Size exclusion chromatography and staining an SDS-PAGE gel with Coomassie Blue shows disassociation of AcsA and AcsB during purification in the presence of LDAO. (b) Purification using buffer containing NaTC caused His-tagged AcsA to remain on the Co-NTA resin. In the SDS-PAGE gel, "Co-NTA" represents the protein eluted from the Co-NTA resin and the numbers indicate the fraction separated by size exclusion chromatography. (PDF) [file pone.0155886.s002.pdf]

S3 Figure

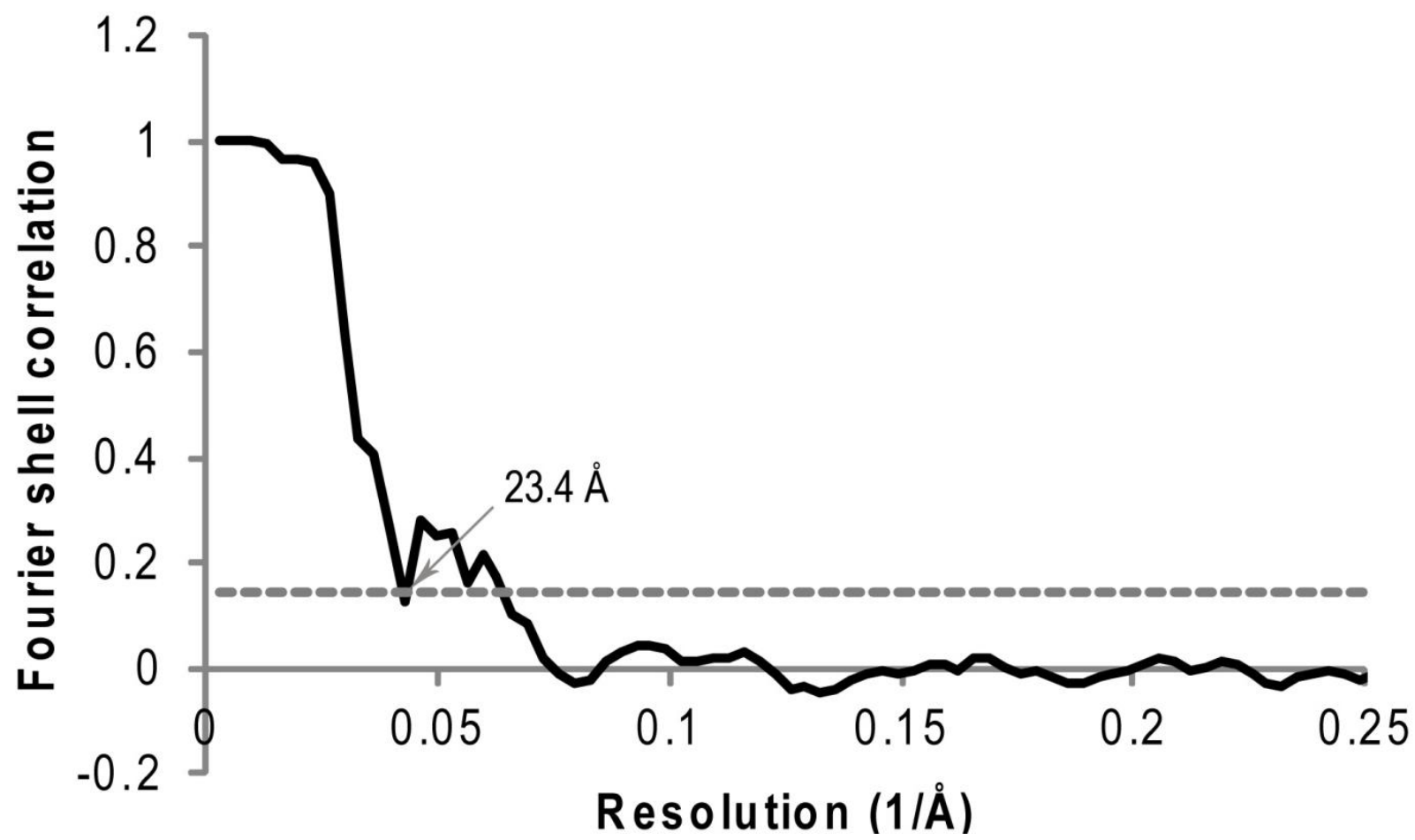

Supplement: S3 Fig — The density map is resolved to 23.4 Å according to the FSC value of 0.143. (PDF) [file pone.0155886.s003.pdf]

S4 Fig

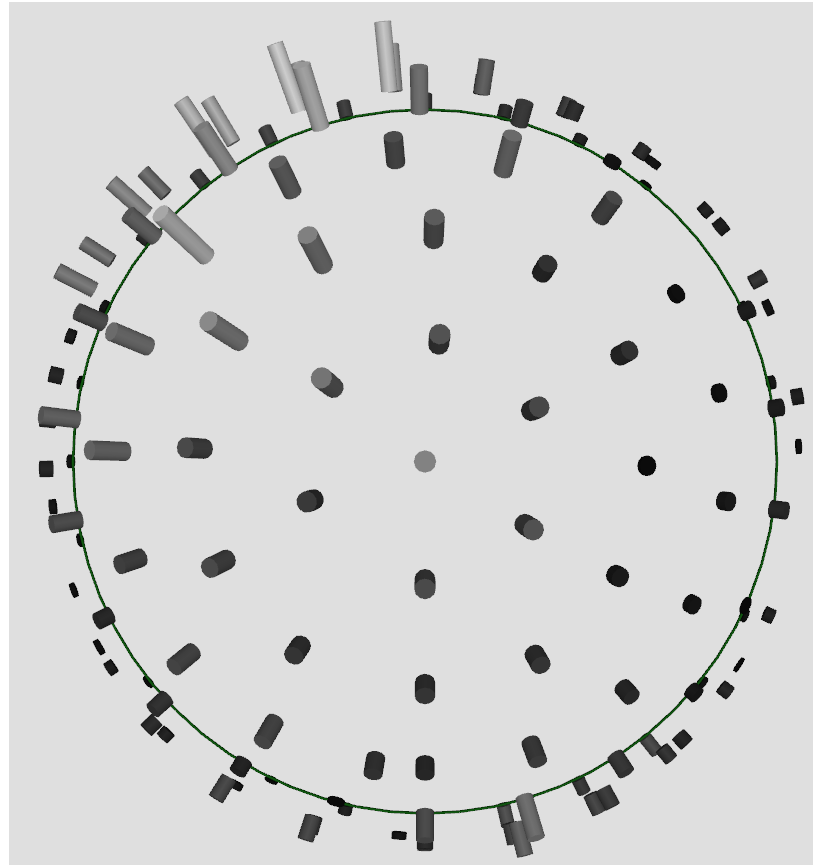

Supplement: S4 Fig — Cylinders of varying height represent the number of particles found in given orientations. Higher and lower cylinders are in light grey and black, respectively. (PDF) [file pone.0155886.s004.pdf]

S5 Fig

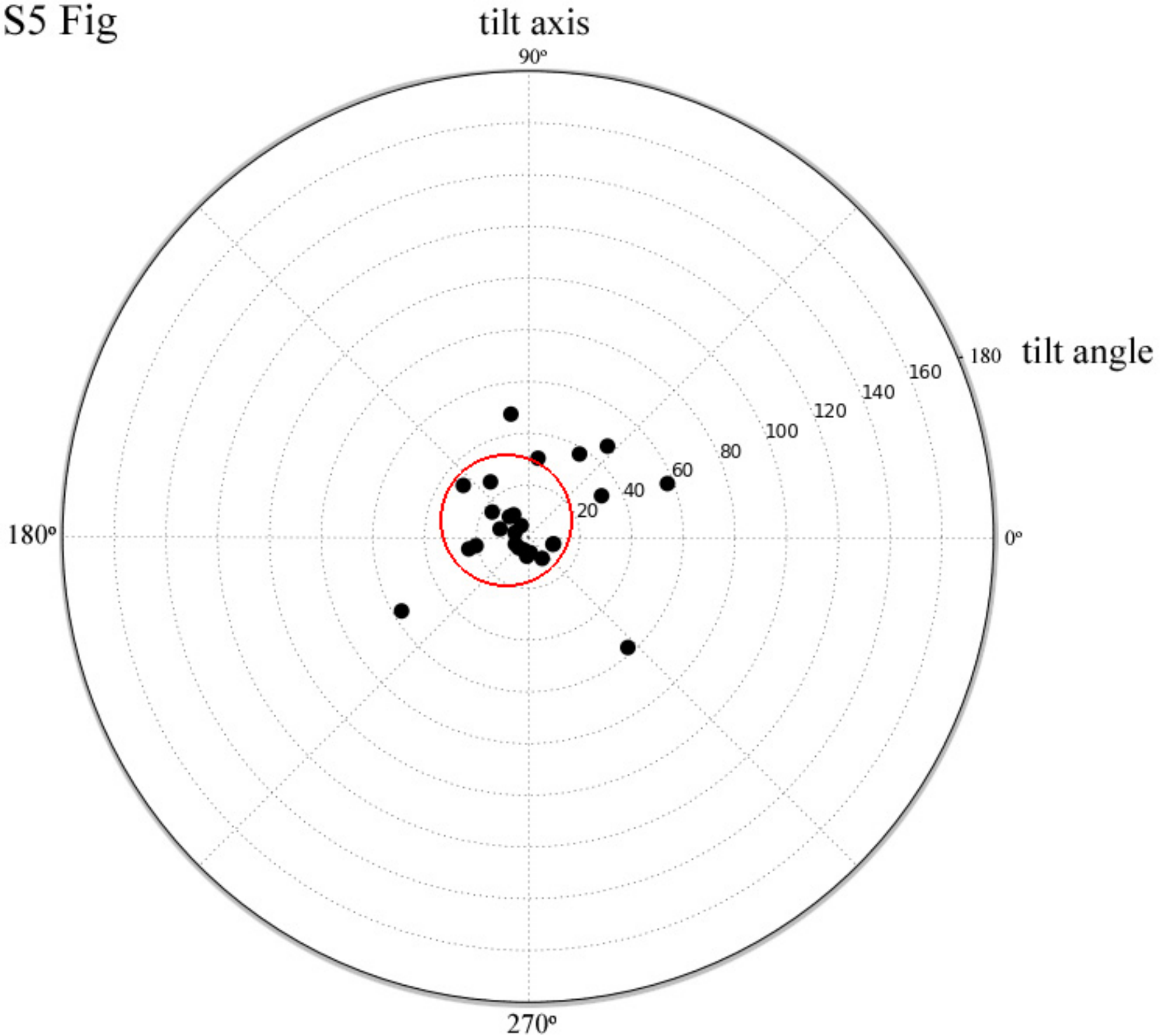

Supplement: S5 Fig — Sixty-eight percent of effective particle pairs cluster around the experimental tilt angle of 10 degrees, though some spread indicates uncertainty in determining particle orientations that is typical of smaller structures. Each black dot represents the tilt axis and tilt angle for a particle pair in polar coordinates. The red circle is centered at the expected relative tilt angle 10°. The outer radius of the plot is 180°. (PDF) [file pone.0155886.s005.pdf]

S6 Fig

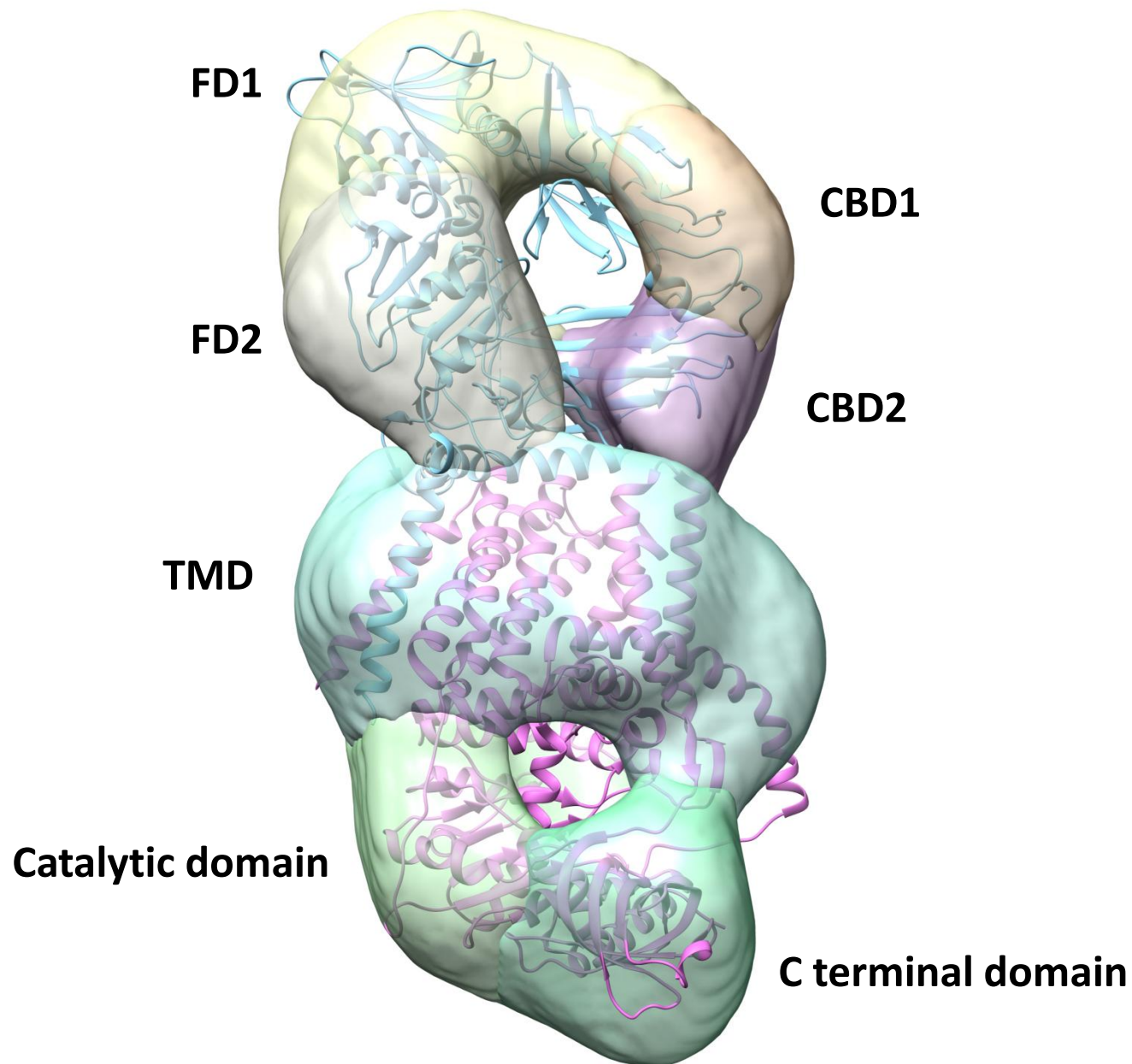

Supplement: S6 Fig — AcsA was segmented into three sub-volumes corresponding to its TM domain (TMD), catalytic domain and C-terminal domain in light blue, light green and springgreen, respectively. AcsB was segmented into four regions: FD1 in yellow, FD2 in violet, CBD1 in orange and CBD2 in purple. (PDF) [file pone.0155886.s006.pdf]

# S7 Figure

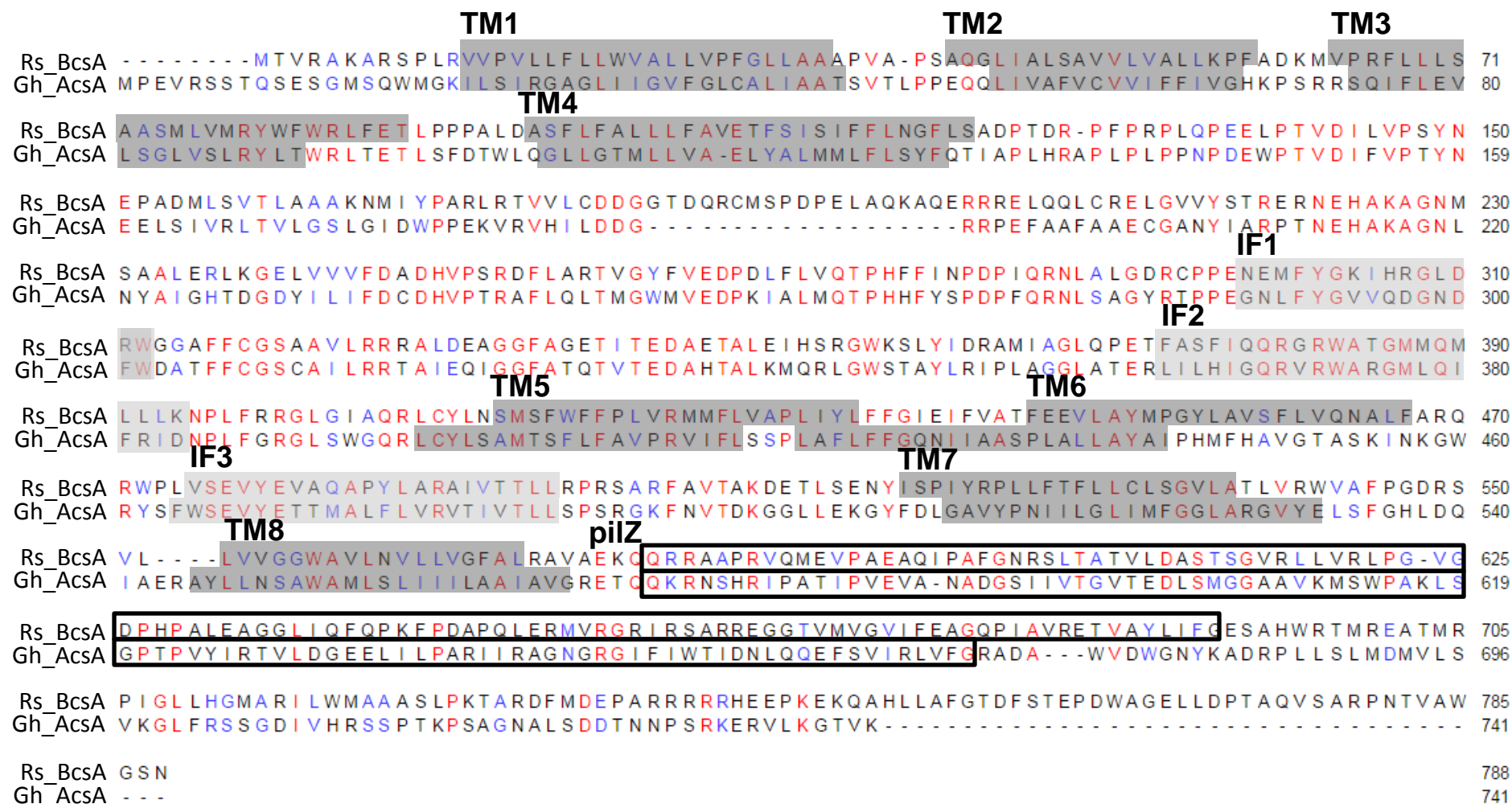

Supplement: S7 Fig — The fused AcsAB sequence is separated into AcsA and AcsB based on previous reports [24, 37]. Secondary structure elements are shown with their corresponding primary amino acid sequences. The major structural domains of BcsA revealed by published crystal structure are shown above the alignment. The conserved transmembrane helices (TMHs), interfacial (IF) helices, and PilZ-domain are indicated with dark grey shade, light grey shade and black box, respectively. (PDF) [file pone.0155886.s007.pdf]

S8 Figure

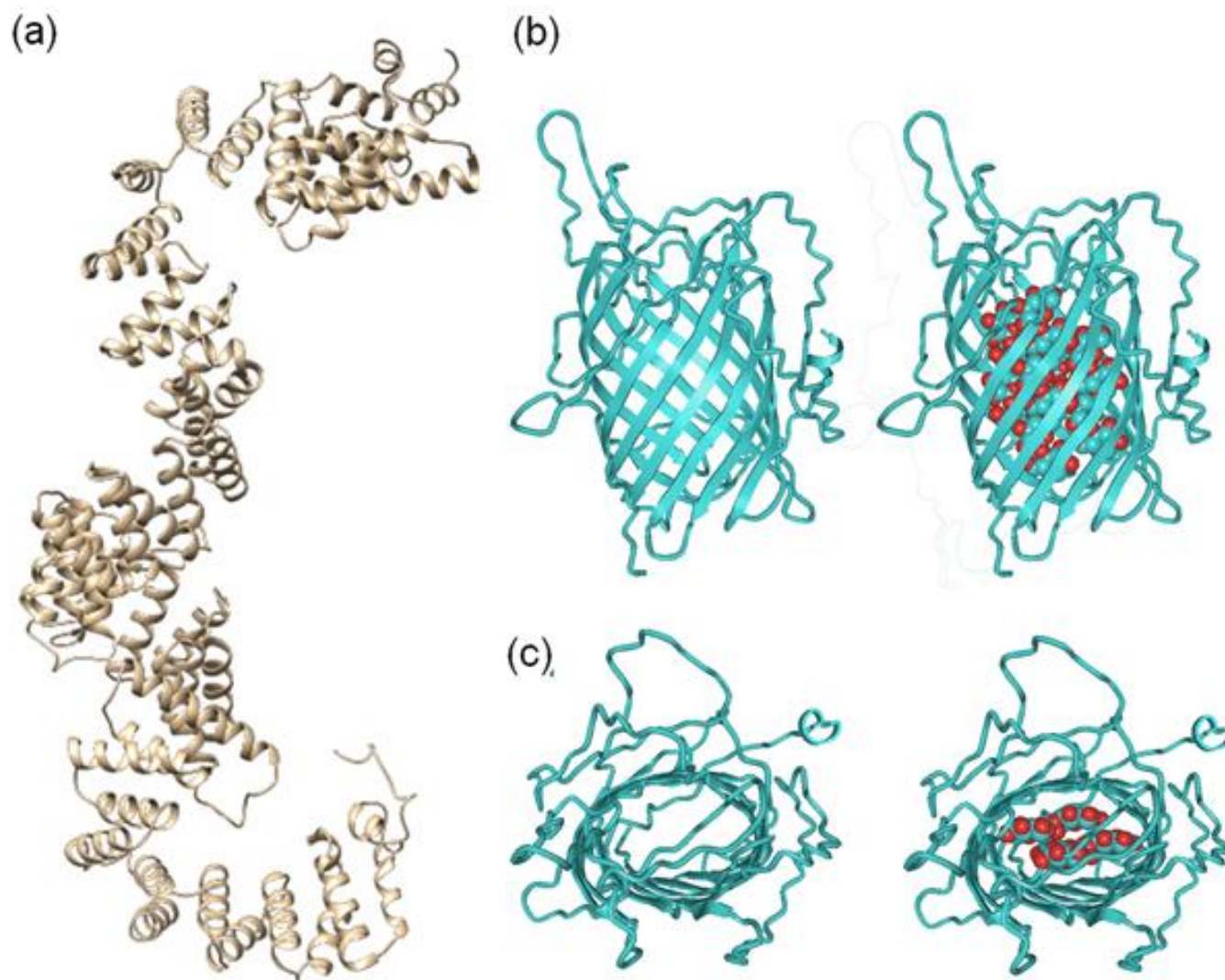

Supplement: S8 Fig — According to the domain prediction of AcsC as shown in S1 Fig, the amino acid sequence of AcsC was split into N-terminal and C-terminal portions. After removing the signal peptide, the N-terminal portion (33-942aa.) and C-terminal portion (943-1302aa.) were separately subjected to structure prediction using ITASSER. (a) Prediction for the N-terminal portion (C-score = 0.53, Estimated TM-score = 0.78±0.09, Estimated RMSD = 7.5±4.3Å). (b) and (c) Prediction for the C-terminal portion (C-score = -3.18) as shown in side view (b) and end view (c), reproduced to the right with 4 strands of cellulose (Iβ crystalline form) inserted into the channel. (PDF) [file pone.0155886.s008.pdf]
