# Supplementary material for: Structure of the Cellulose Synthase Complex of Gluconacetobacter hansenii at 23.4 Å Resolution
Source: PLoS One. 2016 May 23;11(5):e0155886. doi: 10.1371/journal.pone.0155886 (PMC4877109; doi:10.1371/journal.pone.0155886)
Supplement: S1 Table — (PDF) [file pone.0155886.s009.pdf]

S1 Table. Primers used in this study.

Primer name	Forward primer sequence (5'-3')	Reverse primer sequence (5'-3')	Usage
AcsC-T-HR	cgactctagaggatccccgcggaacatcgcatcaata	tgtcaaacatgagaactagtggtgatgatggtgatggtggtgatgatggtgatggccctgaaaatacaggttttctgcccggaataccggacg	C terminal amplification of AcsC-T
AcsC-C-HR	cgactctagaggatccctacgctgggcaataccatt	tgtcaaacatgagaactagtggtgatgatggtgatggtggtgatgatggtgatggccctgaaaatacaggttttctgcccggaataccggacg	C terminal amplification of AcsC-C
Tet	ttctcatgtttgacagcttatca	gacggccagtgaattctcaggtcgaggtggcccggct	Promoter and coding sequence amplification of tetrocycline resistance gene
acs promoter-1	tccttcggtcctccgatcgggccctatattcaggcgac	tcgtttatgggtcacaaacaactcgtccgaaatgc	Amplification of acs operon promoter for driving acsCD
AcsCD	gtgacccataaacgatatgcttcgtc	aagggcatcggtcgactcaggtcgcggaactgcgca	Amplification of AcsC and AcsD coding sequence
acs promoter-2	cgcgacctgagtcgacggccctatattcaggcgac	gtggtgatgatggtgatggtggtgatgatggtgatgctctggcataaacaactcgtccg	Amplification of acs operon promoter for driving acsAB
HisAcsAB	caccatcatcaccacgttcggtcgtcaacgcagtcag	aagggcatcggtcgactcacgacttgcgcctctcatcct	Amplification of AcsAB coding sequence
